# Ferroelectric photosensor network: an advanced hardware solution to real-time machine vision

**DOI:** 10.1038/s41467-022-29364-8

**Published:** 2022-03-31

**Authors:** Boyuan Cui, Zhen Fan, Wenjie Li, Yihong Chen, Shuai Dong, Zhengwei Tan, Shengliang Cheng, Bobo Tian, Ruiqiang Tao, Guo Tian, Deyang Chen, Zhipeng Hou, Minghui Qin, Min Zeng, Xubing Lu, Guofu Zhou, Xingsen Gao, Jun-Ming Liu

**Affiliations:** 1 Institute for Advanced Materials and Guangdong Provincial Key Laboratory of Optical Information Materials and Technology, South China Academy of Advanced Optoelectronics, South China Normal University, Guangzhou, 510006 China; 2 Key Laboratory of Polar Materials and Devices, Ministry of Education, East China Normal University, Shanghai, 200241 China; 3 National Center for International Research on Green Optoelectronics, South China Normal University, Guangzhou, 510006 China; 4 Laboratory of Solid State Microstructures and Innovation Center of Advanced Microstructures, Nanjing University, Nanjing, 210093 China

**Keywords:** Information storage, Ferroelectrics and multiferroics

## Abstract

Nowadays the development of machine vision is oriented toward real-time applications such as autonomous driving. This demands a hardware solution with low latency, high energy efficiency, and good reliability. Here, we demonstrate a robust and self-powered in-sensor computing paradigm with a ferroelectric photosensor network (FE-PS-NET). The FE-PS-NET, constituted by ferroelectric photosensors (FE-PSs) with tunable photoresponsivities, is capable of simultaneously capturing and processing images. In each FE-PS, self-powered photovoltaic responses, modulated by remanent polarization of an epitaxial ferroelectric Pb(Zr_0.2_Ti_0.8_)O_3_ layer, show not only multiple nonvolatile levels but also sign reversibility, enabling the representation of a signed weight in a single device and hence reducing the hardware overhead for network construction. With multiple FE-PSs wired together, the FE-PS-NET acts on its own as an artificial neural network. In situ multiply-accumulate operation between an input image and a stored photoresponsivity matrix is demonstrated in the FE-PS-NET. Moreover, the FE-PS-NET is faultlessly competent for real-time image processing functionalities, including binary classification between ‘X’ and ‘T’ patterns with 100% accuracy and edge detection for an arrow sign with an F-Measure of 1 (under 365 nm ultraviolet light). This study highlights the great potential of ferroelectric photovoltaics as the hardware basis of real-time machine vision.

## Abstract (web summary)

Robust, fast, and low-power hardware platforms are desirable for the implementation of real-time machine vision. Here the authors develop a computing-in-sensor network using ferroelectric photo sensors with remanent-polarization-controlled photo responsivities.

## Introduction

**Fig. 1:**
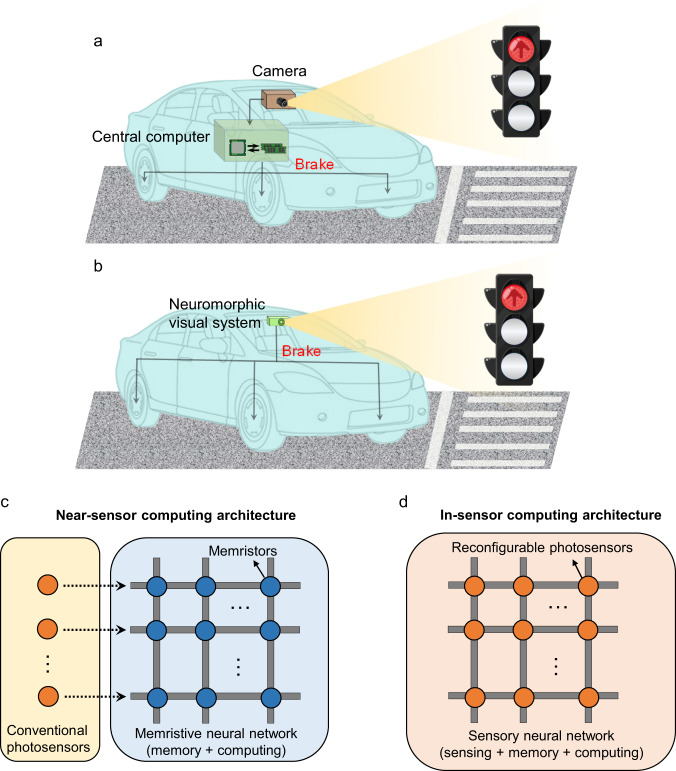
Hardware implementations of machine vision. Schematics for **a** conventional Von Neumann system and **b** emerging neuromorphic visual system. **c** Near- and **d** in-sensor computing architectures for the neuromorphic visual system. The proposed FE-PS-NET uses the architecture shown in **d**, where FE-PSs act as reconfigurable photosensors.

Machine vision is a technology that enables a machine to ‘see’ and ‘understand’ images and videos, which has been widely applied in industry and daily life. In the conventional design of machine vision systems (Fig. 1a), visual information is captured by a photosensor array, converted into electrical digital signals, and passed to a computing unit for image processing^1,2^. The shuttling of redundant data between separated image sensing and processing units can cause high latency and energy consumption, greatly limiting the performance of machine vision in time-critical applications, such as autonomous driving and object tracking. Emerging bio-inspired neuromorphic visual systems (Fig. 1b) provide an opportunity to overcome this limitation^3^. These systems adopt either near- or in-sensor computing architecture (Fig. 1c, d, respectively) to reduce the data shuttling^4^, thus improving the time and energy efficiencies.

So far, a variety of neuromorphic visual systems have been developed for implementing typical image processing functionalities including contrast enhancement, noise suppression, adaptive imaging, recognition, and auto-encoding^5–15^. Among these systems, reconfigurable photosensor network (PS-NET) with an in-sensor computing architecture (Fig. 1d) is of particular interest because it acts on its own as an artificial neural network (ANN) that can simultaneously sense and process images^5,7^. The key building block for reconfigurable PS-NETs is the photosensor with tunable photoresponsivity. Existing designs for such photosensors mainly employed the gating effect in 2D materials^5,7^ and the ion migration in memristive materials^11,12,15^ to realize the tunable photoresponsivity. However, the required application of gate voltage inevitably consumes additional powers^16^, while the ion migration is kinetically slow and the ion relaxation may cause poor retention^11,17^. New types of reconfigurable photosensors with improved speed, energy efficiency, and reliability are therefore highly desirable.

Ferroelectric photosensor (FE-PS) emerges as an advanced reconfigurable photosensor with all above desired performance. Using the remanent polarization to tune the photovoltaic response^18–23^, the FE-PS is essentially a gate voltage-free and self-powered reconfigurable photosensor^24–28^. Notably, the polarization switching can induce not only the magnitude change but also the sign reversal of photoresponse^19,21^, enabling a single FE-PS to represent both positive and negative weights and hence reducing the hardware overhead for network construction. Moreover, the nonvolatility, high controllability, and ultrafast switching kinetics (<1 ns) of polarization as demonstrated in various ferroelectric memory and neuromorphic devices^29–34^, along with the intimate coupling between polarization and photoresponse^35^, endow the FE-PS with good reliability and high write speed. Also noteworthy are the high photosensitivity and ultrashort photoresponse time (<1 ns) of FE-PS^24,25^, allowing a high-speed readout. Given the above merits of FE-PS, the FE-PS network (FE-PS-NET), a computing-in-sensor circuit built with interconnected multiple FE-PSs (Fig. 1d), appears very promising as a fast, low-power, and reliable hardware solution to real-time machine vision. However, while the ferroelectric neuromorphic devices with the memory-computing integrated paradigm have been extensively investigated recently, the FE-PS-NET, representing the first extension to the sensing-memory-computing integrated paradigm, remains experimentally unexplored yet.

Here, we demonstrate a prototype FE-PS-NET with integrated image sensing and processing functions. Each FE-PS in the network consists of a Pt/Pb(Zr_0.2_Ti_0.8_)O_3_ (PZT)/SrRuO_3_ (SRO) heterostructure epitaxially grown on a SrTiO_3_ (STO) substrate. The high-quality epitaxial PZT film is chosen as the ferroelectric layer for FE-PS because of its large remanent polarization as well as strong and highly controllable photoresponse [albeit in the ultraviolet (UV) spectrum]^21,36^. SRO is used as the bottom electrode and it also facilitates the epitaxial growth of PZT. The fabricated PZT-based FE-PS exhibits symmetrically switchable, nonvolatile, and multilevel photovoltaic responses as controlled by the remanent polarization. These unique properties enable the FE-PS to be a highly reliable and self-powered reconfigurable photosensor capable of exhibiting both positive and negative photoresponsivities (i.e., weights). Multiple individual FE-PSs are then wired into an FE-PS-NET (see photos in Supplementary Fig. S1), whose capability to perform an in situ multiply-accumulate (MAC) operation between an input image and a photoresponsivity matrix is experimentally evidenced. The FE-PS-NET is further used to implement real-time image processing functionalities, including binary pattern classification with 100% accuracy and edge detection with an F-Measure of 1. Moreover, the ultralow latency and zero energy consumption for inference are prospected for the FE-PS-NET, underscoring its potential as a hardware platform for real-time machine vision.

## Results

### Tunable nonvolatile photoresponsivity in FE-PS

**Fig. 2:**
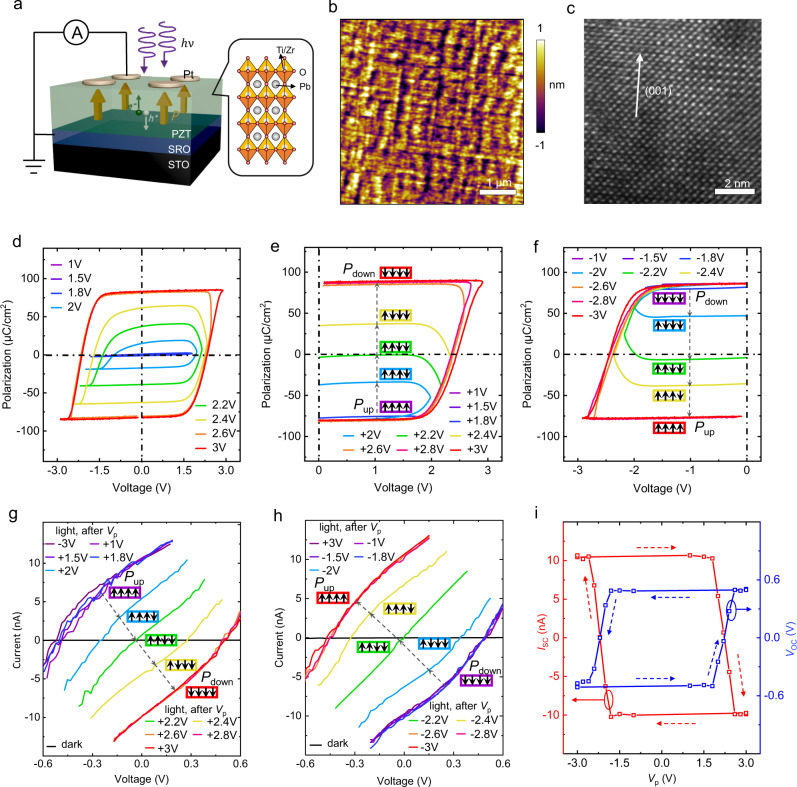
Polarization switching behavior and polarization-tuned multilevel nonvolatile photovoltaic responses in FE-PS. **a** Schematics illustrating the device structure of the Pt/ PZT (epitaxial film)/SRO FE-PS (left panel) and the crystal structure of PZT (right panel). **b** AFM topography image and **c** high-resolution cross-sectional TEM image of the epitaxial PZT film. **d** Bipolar, **e** positive monopolar, and **f** negative monopolar *P–V* hysteresis loops measured with different pulse voltages. Illuminated *I–V* characteristics measured after applying different **g** positive pulses and **h** negative pulses to the FE-PS initialized by −3 V and +3 V pulses (pulse width: 0.15 ms), respectively. In **e**–**h**, the different polarizations states are schematically illustrated by the configurations of the four black solid arrows. **i** *I*_sc_ and *V*_oc_ as a function of the write pulse voltage (*V*_p_).

The designed FE-PS has a simple two-terminal structure of Pt/PZT/SRO, as schematically illustrated in Fig. 2a. The PZT/SRO bilayer film was epitaxially grown on the STO (001) substrate by pulsed laser deposition (PLD). The thickness of PZT layer was controlled to be ~120 nm, the reason for which is given in Supplementary Note 1. The Pt top electrodes were deposited ex situ by PLD through a shadow mask (diameter: ~200 μm). The X-ray diffraction (XRD) and transmission electron microscopy (TEM) results of the fabricated PZT/SRO/STO heterostructure are shown in Supplementary Fig. S2, revealing the epitaxial growth of both PZT (~120 nm) and SRO (~40 nm) layers with typical perovskite phases. The atomic force microscopy (AFM) image of the PZT/SRO film shows a flat surface with a small root-mean-square roughness of ~470 pm (Fig. 2b). The high-magnification cross-sectional TEM image further reveals the well-aligned lattice of PZT (Fig. 2c). These characterizations demonstrate the high quality of the epitaxial PZT film, which is a prerequisite for obtaining good ferroelectric and photovoltaic properties.

The ferroelectric properties of the Pt/PZT/SRO device were investigated by measuring the bipolar and monopolar polarization-voltage (*P–V*) hysteresis loops using triangular pulses (pulse width: 0.15 ms). The voltage was applied to the Pt electrode with the SRO electrode grounded. Figure 2d shows the pulse voltage (*V*_p_)-dependent bipolar *P–V* loops. The loop starts to open as *V*_p_ exceeds 1.8 V and becomes almost saturated when *V*_p_ reaches 2.6 V. The saturated *P–V* loops reveal a large remanent polarization of ~80 μC/cm^2^, a typical polarization value of high-quality epitaxial PZT films^21,36,37^. Another key feature of the saturated loops is the negligible voltage offset, namely, the positive and negative coercive voltages are almost symmetric, suggesting that there is only small or even no imprint field. Because the imprint field often originates from the defects^38,39^, its absence in turn verifies the high quality of our epitaxial PZT film. Besides, the absence of imprint field contributes to the symmetry of switchable photovoltaic responses^38,40^ (to be shown later).

Figure 2d also displays that multiple intermediate polarization states are accessible when *V*_p_ is in the range of 1.8–2.6 V. To confirm it, monopolar triangular pulses with different *V*_p_ were applied and the measured *P–V* loops are shown in Fig. 2e, f. Every time before applying the measurement pulse, a −3 V or +3 V preset pulse was applied to set the complete polarization up (*P*_up_) or down (*P*_down_) state, respectively. As seen from Fig. 2e, when starting from the same complete *P*_up_ state (~−80 μC/cm^2^), applying positive pulses with *V*_p_ ≤ +1.8 V makes almost no change in the polarization state. Applying positive pulses with *V*_p_ = +2, +2.2, and +2.4 V results in three well-separated intermediate states: incomplete *P*_up_, near-zero-polarization, incomplete *P*_down_ states, whose corresponding remanent polarization values are ~−40, ~0, and ~+40 μC/cm^2^, respectively. Further increasing *V*_p_ to +2.6 V and above switches the device to the complete *P*_down_ state (~+80 μC/cm^2^). Likewise, by applying negative pulses with increasing *V*_p_, the complete *P*_down_ state is switched to the incomplete *P*_down_ state, near-zero-polarization state, incomplete *P*_up_ state, and eventually complete *P*_up_ state (Fig. 2f). In addition, the loops in Fig. 2e and f exhibit relatively flat tops and bottoms, respectively, indicating that the polarizations can be retained when the external voltages return to zero. This implies that the polarization states, including the intermediate states, are nonvolatile.

The formation mechanism of intermediate polarization states was investigated by using piezoresponse force microscopy (PFM). Supplementary Fig. S3 shows that the downward (upward) domains can be gradually switched upward (downward) as the applied negative (positive) tip voltage increases. In particular, upward/downward mixed domain configurations are observed when medium tip voltages are applied, giving rise to intermediate polarization states. All the domain states are found to be stable (up to 18 days of retention), confirming the nonvolatility of the polarization states. Such good domain stability may benefit from the domain growth-dominated switching behavior, as discussed in Supplementary Fig. S3.

**Fig. 3:**
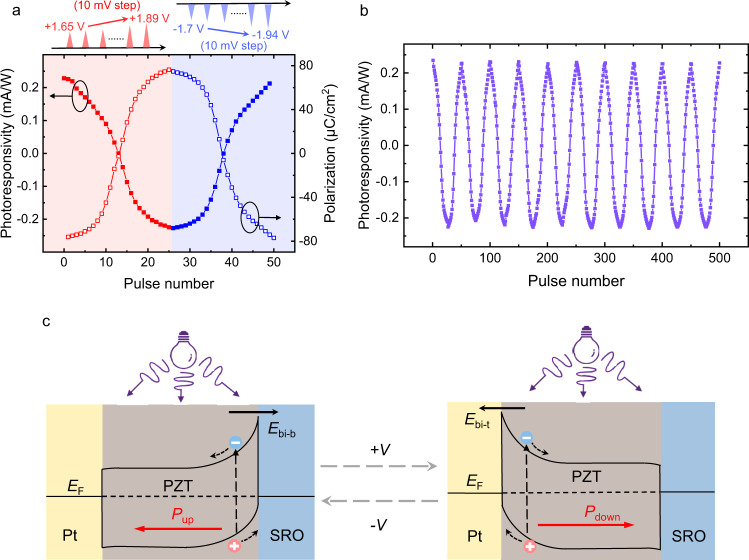
Synaptic behavior and operation mechanism of FE-PS. **a** One-cycle LTD and LTP characteristics where the photoresponsivity represents the weight along with the evolution of polarization during the pulse application. Upper panel shows the schematics of the positive and negative pulses applied in succession, while the preset pulse applied at the very beginning is not shown. **b** Multi-cycle LTD and LTP characteristics. **c** Schematic energy band diagrams of the FE-PS in the complete *P*_up_ (left panel) and *P*_down_ (right panel) states. The polarization switching can tune the Schottky barrier heights and built-in fields at the two interfaces, thus modifying the magnitude and direction of the overall output photocurrent.

Given the excellent ferroelectric properties of the present epitaxial PZT film (including large remanent polarization, negligible voltage offset, and accessibility to multiple nonvolatile polarization states), the polarization-modulated photovoltaic behavior in the PZT-based FE-PS is worthy of investigation. To characterize it, monopolar triangular pulses were applied first to write the polarization states, and every time before applying a write pulse the preset pulse was applied, as illustrated in Fig. 2e, f. In each polarization state, current-voltage (*I–V*) characteristics under illumination were recorded by using the 365 nm UV light for illumination, because this light wavelength corresponds well to the bandgap of PZT (~3.6 eV)^36^. Unless otherwise specified, the applied light intensity was ~150 mW/cm^2^ (the corresponding optical power was ~47.1 μW for an electrode area of ~0.0314 mm^2^). Figure 2g shows the illuminated *I–V* curves of the FE-PS in the different polarization states as set by the different positive pulses. In the initial −3 V-written state, the FE-PS exhibits noticeable photovoltaic responses including a short-circuit current (*I*_sc_) of ~10.6 nA and an open-circuit voltage (*V*_oc_) of ~−0.5 V. Such *I*_sc_ is three orders of magnitude larger than the dark current (~−0.02 nA at −0.5 V). The illuminated *I–V* curve, as well as *I*_sc_ and *V*_oc_, remains almost unchanged after applying positive pulses with *V*_p_ ≤ +1.8 V. After applying the +2 V pulse, the illuminated *I–V* curve shifts toward the origin, and *I*_sc_ and *V*_oc_ decrease to ~5.4 nA and ~−0.25 V, respectively. As *V*_p_ increases to +2.2 V, the illuminated *I–V* curve moves very close to the origin, showing near-zero values of *I*_sc_ (~0.7 nA) and *V*_oc_ (~−0.04 V). Increasing *V*_p_ to +2.4 V pushes the illuminated *I–V* curve away from the origin along the positive voltage and negative current axes. As a result, both *I*_sc_ and *V*_oc_ change their signs (i.e., directions), and their values are ~−4.4 nA and ~0.28 V, respectively. The illuminated *I–V* curve is further pushed away from the origin after applying the +2.6 V pulse, and *I*_sc_ and *V*_oc_ become ~−9.9 nA and ~0.5 V, respectively. Further increasing *V*_p_ to +2.8 V and above makes no more change in the illuminated *I–V* curve. There are thus five photoresponsive states observed during the switching process (note: a much larger number of states are indeed accessible, to be shown in Fig. 3). Apparently, these photoresponsive states show almost one-to-one correlation with the polarization states (Fig. 2e), demonstrating that the photoresponse is well controlled by the polarization.

In addition, the reverse switching of the photoresonsive state is observed by applying negative pulses (Fig. 2h), which is consistent with the down-to-up polarization switching (Fig. 2f). Plots of *I*_sc_ and *V*_oc_ against the pulse voltage *V*_p_ form well-shaped hysteresis loops akin to the *P–V* hysteresis loops (Fig. 2i), further confirming the reversible polarization control of the photovoltaic response. Tunable photoresponse obtained in the photovoltaic mode qualifies the FE-PS as a self-powered reconfigurable photosensor. In addition, unlike previous photovoltaic-type reconfigurable photosensors^5^, the FE-PS does not need to use the gate voltage to switch the photoresponse. It uses the remanent polarization as the control knob instead, which can further lower the power consumption.

Figure 2g, h also show that in a pair of *P*_up_ and *P*_down_ states with opposite remanent polarizations, the two *I*_sc_ (or *V*_oc_) values have opposite signs but similar magnitudes. For example, the *I*_sc_ value in the complete *P*_up_ state is ~10 nA, while that in the complete *P*_down_ state is just the opposite, i.e., ~−10 nA. Such symmetry of switchable photoresponses is a result of the dominated polarization control, which benefits from the high-quality epitaxial PZT film possessing large switchable polarization and negligible imprint field (see evidence from the symmetric *P–V* loops in Fig. 2d)^38,40^. From the application point of view, symmetrically switchable photoresponses enable a single FE-PS to represent both positive and negative weights, which is particularly useful for reducing the number of FE-PSs needed for network construction.

Because the photovoltaic behavior is controlled by the polarization without involving defect-mediated mechanisms (e.g., ion migration), good reliability is expected for FE-PS. We first investigated the photoresponse stability. As shown in Supplementary Fig. S4, the polarization-controlled photoresponsive states are stable with a rather long retention time of ≥24 h, and they are reproducible during the frequent ON/OFF illumination cycling. This demonstrates the nonvolatility of the photoresponsive states, which benefits from the nonvolatility of the polarization states (Fig. 2d–f and Supplementary Fig. S3). Then, the endurance test was performed by switching the FE-PS with cyclic 3 V/10 μs pulses. Supplementary Fig. S5 shows that both the photocurrents and associated remanent polarizations change only slightly after switching for 10^6^ cycles, highlighting the good endurance of the FE-PS. Device-to-device variation was characterized by measuring the *P–V* loops and photocurrents of 11 different FE-PSs (Supplementary Fig. S6). These devices all exhibit switchable photocurrents, and the photocurrents in the same polarization state show a small variation of ~3.2%. In addition, to enable the FE-PS to perform the multiplication (i.e., photosensing) reliably, a linear dependence of photocurrent on light intensity is required. As shown in Supplementary Fig. S7, the photocurrents in different polarization states scale almost linearly with the light intensity (linearity: ≥0.94), thus satisfying the requirement of multiplication.

The polarization control of photovoltaic behavior with high reliability promises our FE-PS as a superior synaptic device (using the photoresponsivity as the weight). To demonstrate it, typical synaptic behaviors, i.e., long-term potentiation and depression (LTP and LTD, respectively), were measured for the FE-PS. In the measurement, the FE-PS was initialized in the complete *P*_up_ state by applying a −3 V/0.15 ms preset pulse. Then, 25 positive triangular pulses (amplitude: from 1.65 V to 1.89 V in increments of 0.01 V; width: 10 μs) and 25 negative triangular pulses (amplitude: from −1.7 V to −1.94 V in decrements of 0.01 V; width: 10 μs) were applied successively without preset pulses inserted between them (see upper panel in Fig. 3a). The pulse voltages were slightly below the coercive voltages and increased in magnitude so that the polarization could be switched gradually to produce many intermediate states (note: the coercive voltages of the device used for the LTP/LTD measurement are ~±1.9 V and it is confirmed that the applied pulses can gradually switch the polarization; see Supplementary Fig. S8–S12 for details). After each positive or negative pulse, *I*_sc_ was measured and used to calculate the photoresponsivity defined as

1 $$R={I}_{{{{{{\rm{sc}}}}}}}/P,$$

where *R* is the photoresponsivity and *P* is the input optical power (product of light intensity and electrode area). *R* is a signed quantity because the *I*_sc_ values in the different polarization states can have different signs (Fig. 2g–i).

As shown in Fig. 3a, *R* decreases gradually from ~0.22 mA/W to ~−0.22 mA/W with increasing the number of positive pulses, indicating the LTD behavior. By contrast, *R* increases from ~−0.22 mA/W back to ~0.22 mA/W under the stimulation of negative pulses, a manifestation of the LTP behavior. The corresponding systematic shift of the illuminated *I–V* curve is shown in Supplementary Fig. S13. Figure 3a also reveals that the gradual evolution of *R* from positive maximum to negative maximum and back to positive maximum is well consistent with the gradual polarization switching from *P*_up_ to *P*_down_ and back to *P*_up_, further confirming the polarization control of *R*.

Similar LTD and LTP characteristics can be reproduced for many cycles (Fig. 3b), showing a small cycle-to-cycle variation of ~3%. Notably, each LTD or LTP process contains 25 different *R* levels, confirming the accessibility to multiple photoresponsive states. One may further achieve a larger number of *R* levels by manipulating the applied pulses.

The integrated synaptic and photosensing functions of the FE-PS, as demonstrated above, allow the construction of FE-PS-NET with in-sensor computing capability. Prior to constructing it, the physical mechanism underlying the polarization control of photovoltaic behavior in the FE-PS needs to be understood. We previously demonstrated that the polarization-modulated Schottky barrier was responsible for the switchable photovoltaic behavior in the Pt/PZT/SRO FF-PS, through comprehensive investigations on the ferroelectric, dielectric, conduction, and photovoltaic behaviors of the device^36^. In brief, epitaxial PZT film which is an *n*-type semiconductor^41^ can form Schottky barriers with Pt and SRO. Assuming that there is no polarization in PZT, the top Pt/PZT and bottom PZT/SRO barriers would have similar heights due to the similar work functions of Pt and SRO (~5.3 and ~5.2 eV, respectively). However, the polarization of PZT can significantly modify the barrier heights at the top and bottom interfaces as well as the associated built-in fields (*E*_bi-t_ and *E*_bi-b_, respectively). In the complete *P*_up_ state, the negative polarization charge at the PZT/SRO interface enhances the bottom barrier height and *E*_bi-b_, while the positive polarization charge at the Pt/PZT interface reduces (or even eliminates) the top barrier height and *E*_bi-t_ (see the left panel of Fig. 3c)^42,43^. The downward *E*_bi-b_ therefore dominates and generates an overall positive photocurrent. By contrast, the dominance of *E*_bi-t_ occurs in the complete *P*_down_ state, producing an overall negative photocurrent (see the right panel of Fig. 3c). In the intermediate polarization states, the relative proportion of upward and downward domains may determine the magnitude and direction of overall photocurrent, and hence multilevel photocurrents are accessible. The Schottky barrier modulation can therefore well explain the polarization-controlled switchable photoresponse in the Pt/PZT/SRO FE-PS.

### In-sensor MAC operations in FE-PS-NET

**Fig. 4:**
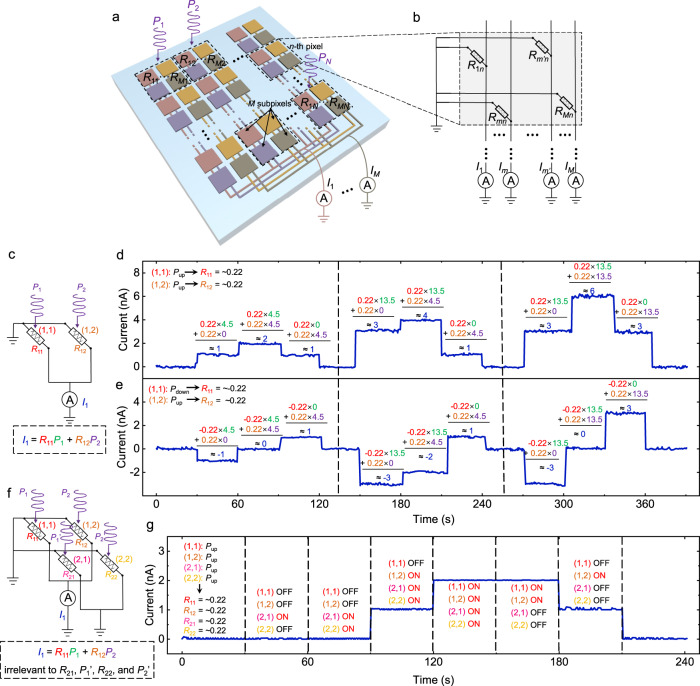
In-sensor MAC operations in FE-PS-NET. **a** Schematic illustration of the architecture of the FE-PS-NET. **b** Schematic circuit diagram for a pixel in the FE-PS-NET. **c** Schematic circuit diagram for a 1 × 2 FE-PS-NET. **d**, **e** Time-resolved currents (*I*_1_) measured during the applications of different illuminations to the (1, 1) and (1, 2) FE-PSs. In **d** the (1, 1) and (1, 2) FE-PSs are both set in the complete *P*_up_ state, while in e the two devices are set in the complete *P*_down_ and *P*_up_ states, respectively. In each period during which at least one FE-PS is illuminated, the MAC operation is directly expressed as the equation containing the experimental values of photoresponsivity, optical power, and output current. Their corresponding units are mA/W, μW, and nA, respectively (not shown). **f** Schematic circuit diagram for a 2 × 2 FE-PS-NET. **g** Time-resolved currents (*I*_1_) measured during the applications of different illuminations to the (1, 1), (1, 2), (2, 1), and (2, 2) FE-PSs. All the FE-PSs are set in the complete *P*_up_ state, and the illumination sequences are indicated in the corresponding periods while the optical power for illumination (~4.5 μW) is not shown.

Having demonstrated the switchable photoresponsivity of FE-PS and understood its physical mechanism, it is of interest to investigate the hardware implementation of MAC (a fundamental operation for the simultaneous image sensing and processing) using FE-PS-NET. As schematically shown in Fig. 4a, b, the FE-PS-NET consists of *N* pixels with each pixel divided into *M* subpixels. *N* depends on the image size, i.e., *N* = *H* × *W*, where *H* and *W* are the height and width of the image, respectively, and the *N* pixels are arranged in an *H* × *W* array to suit the image. The *M* subpixels are also arranged in a 2D array for saving the area overhead. Each subpixel corresponds to an FE-PS, which has a subpixel index (*m* = 1, 2,…, *M*) as well as a pixel index (*n* = 1, 2,…, *N*). The FE-PSs with the same subpixel index *m* are connected in parallel (for inference only; Supplementary Fig. S14 for more descriptions). With such an architecture, the FE-PS-NET can perform an efficient in-sensor MAC operation: under short-circuit and illumination conditions, the multiplication of optical power and photoresponsivity occurs at each individual FE-PS through the photosensing process; meanwhile, the photocurrents generated by the *N* FE-PSs with the same subpixel index *m* are summed together according to the Kirchhoff’s law. The output current *I*_*m*_ is expressed as

2 $${I}_{m}=\mathop{\sum }\limits_{n=1}^{N}{R}_{mn}{P}_{n},$$

where *R*_*mn*_ is the photoresponsivity of the FE-PS at the *n*-th pixel and *m*-th subpixel [denoted as the (*m*, *n*) FE-PS hereafter], and *P*_*n*_ is the input optical power at the *n*-th pixel [the vector ***P***_**in**_ = (*P*_1_, *P*_2_,…, *P*_*N*_)^T^ represents the input image].

To experimentally demonstrate the MAC, i.e., Eq. (2), a simple 1 × 2 (*M* = 1 and *N* = 2) FE-PS-NET was used first (Fig. 4c). Both of the two FE-PSs were set in the complete *P*_up_ states beforehand. The resulting photoresponsivities *R*_11_ and *R*_12_ were thus almost the same, i.e., ~0.22 mA/W. As shown in Fig. 4d, during the first 30 seconds, no illumination is applied and hence the output current, i.e., *I*_1_, is observed to be zero. During the period of 30–60 s, the (1, 1) FE-PS is illuminated with an optical power of *P*_1_ = ~4.5 μW, resulting in a photocurrent of ~1 nA. During the next 30 s, both two FE-PSs are illuminated with *P*_1_ = *P*_2_ =  ~4.5 μW. The output current jumps to ~2 nA, which is just the summation of the photocurrents generated by the two FE-PSs. Then, the illumination on the (1, 1) FE-PS is turned off while that on the (1, 2) FE-PS remains. The output current drops to ~1 nA, which is the photocurrent generated by the individual (1, 2) FE-PS. After this, the illuminations on both two FE-PSs are turned off, and consequently the output current returns to zero. During the periods of 150–240 s and 270–360 s, another two rounds of illuminations are applied. These two rounds of illuminations have the same sequence of applying *P*_1_ and *P*_2_ as the first round (30–120 s). However, the magnitudes of *P*_1_ and *P*_2_ are adjusted: *P*_1_ = ~13.5 μW and *P*_2_ = ~4.5 μW in the second round while *P*_1_ = ~13.5 μW and *P*_2_ = ~13.5 μW in the third round. Inspecting the periods where only one individual FE-PS is illuminated, one can find that the photocurrent of the individual FE-PS scales with the optical power, confirming the validity of multiplication. Besides, it is observed that the output currents during the periods where both two FE-PSs are illuminated always equal the summed photocurrents of the two FE-PSs.

After these measurements, the (1, 1) FE-PS was set in the complete *P*_down_ state while no change of polarization state was made for the (1, 2) FE-PS. The resulting photoresponsivities *R*_11_ and *R*_12_ were thus ~−0.22 mA/W and ~0.22 mA/W, respectively. Then, three rounds of illuminations same as those used in Fig. 4d were applied again to the 1 × 2 FE-PS-NET, and the output currents are shown in Fig. 4e. The photocurrent generated by the (1, 1) FE-PS is observed to be negative, well attributed to the negative *R*_11_. Moreover, both multiplication and summation operations are observed to be valid. The combined Fig. 4d, e therefore demonstrate that the 1 × 2 FE-PS-NET can perform the MAC operations following Eq. (2).

Whether Eq. (2) still applies in a FE-PS-NET with larger size remains a question because the sneak path issue may arise. The sneak path issue refers to the unintentional current flow through neighboring unselected devices in a crossbar structure. It is a common issue encountered by conventional memristor crossbar-based ANNs^44^, which can cause significant errors in output currents. The sneak path issue in our FE-PS-NET was investigated with a 2 × 2 (*M* = 2 and *N* = 2) FE-PS-NET, as schematically shown in Fig. 4f. All the four FE-PSs were set in the complete *P*_up_ state, resulting in almost the same photoresponsivity of ~0.22 mA/W. The output current *I*_1_ was monitored while applying a sequence of illuminations to the four FE-PSs. The illuminations for the four FE-PSs were applied or terminated independently, and the optical power during illumination was ~4.5 μW. As seen in Fig. 4g, applying or removing illuminations to the (2, 1) and (2, 2) FE-PSs have almost no influences on the multiplication and summation operations performed by the sub-circuit composed of the (1, 1) and (1, 2) FE-PSs. Therefore, our FE-PS-NET has good immunity to the sneak path issue. The reason for this may be because the FE-PS-NET works under the short-circuit condition and the illumination rather than the bias is used to select the device. The photocurrent generated by a selected device would therefore not flow through a neighboring unselected device. Even in the case where the short-circuit condition is not strictly met and a small bias arising from the photovoltaic effect of a selected device does drop across a neighboring unselected device, the leakage current produced by the neighboring unselected device would be negligible due to its high resistance (Fig. 2g, h). This is fundamentally different from the scenario in a memristor crossbar, where the neighboring unselected device in the ON state can contribute a large leakage current. Despite the good immunity of FE-PS-NET to the sneak path issue demonstrated here, whether the sneak path issue will arise in a practical large-scale network deserves further investigation.

### Implementations of pattern classification and edge detection

**Fig. 5:**
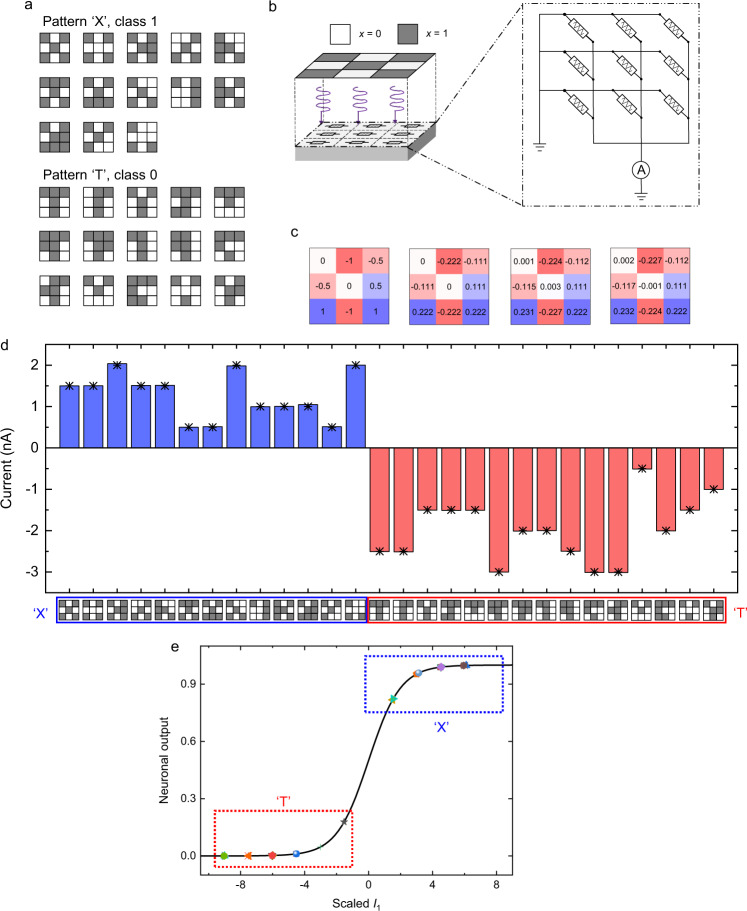
Implementation of pattern classification. **a** Two sets of patterns representing letters ‘X’ and ‘T’. **b** Schematic diagrams showing the operation and the circuit structure of a 1 × 9 FE-PS-NET. The 9 FE-PSs are illuminated following the signals translated from the input pattern. **c** From left to right: theoretical dimensionless weights, theoretical photoresonsivities scaled from dimensionless weights, actual photoresonsivities right after programming, actual photoresonsivities after pattern classification. The unit of photoresponsivity is mA/W (not shown). **d** Output currents (*I*_1_) during the presentations of different input patterns. The theoretical *I*_1_ values are indicated by the “star” symbols. **e** Neuronal outputs obtained by feeding the output currents to the sigmoid function.

With the capability to perform in-sensor MAC operations, the FE-PS-NET can readily be used to implement real-time image processing functionalities. Pattern classification was demonstrated first. Two sets of patterns, representing the letters ‘X’ and ‘T’ and their variants after adding noises (Fig. 5a), were used as both training and test sets^45,46^. Each pattern contained 3 × 3 = 9 pixels, and the pixel values of black and white pixels were defined as 1 and 0, respectively. The classes of ‘X’ and ‘T’ corresponded to the binary outputs of 1 and 0, respectively. Such pattern classification task was solvable by a single-layer perceptron containing nine input neurons and one output neuron. The single-layer perceptron was hardware implemented with a 1 × 9 (*M* = 1 and *N* = 9) FE-PS-NET (Fig. 5b). When presenting an input pattern to the FE-PS-NET, the pixel value of 1 (or 0) at a specific pixel represented applying (or removing) illumination with an optical power of ~4.5 μW to the corresponding FE-PS. Through the MAC process, the FE-PS-NET produced an output current *I*_1_, which was then fed to a sigmoid activation function to generate a neuronal output (Methods). The output current *I*_1_ on the order of several nanoamperes might be small, but it could be amplified using appropriate amplifying circuits^47^ before being fed to the sigmoid activation function. The sigmoid activation function was implemented in software here, but it could be implemented with conventional CMOS circuits^48^. The training was also performed in software, a method called the ex-situ training. Then, the calculated weight matrix was transferred to the FE-PS-NET. When programming each FE-PS, a write-and-verify method was used to ensure a small discrepancy between the actual and target photoresponsivities. The FE-PS-NET after programming could conduct the inference once an input pattern was presented to it.

Figure 5c compares the calculated and actual weight matrices. The differences between the actual weights and the corresponding calculated ones are quite small, indicating the successful programming of the FE-PS-NET. Figure 5d shows the output current *I*_1_ during the presentations of different input patters. It is observed that the output current is always positive when a pattern belonging to the ‘X’ class is presented, while it is always negative when a pattern belonging to the ‘T’ class is presented. Moreover, the measured output currents agree well with the theoretically calculated ones. Figure 5e further presents the neuronal outputs derived from the output currents. The neuronal outputs of the ‘X’-class patterns are all close to 1 while those of the ‘T’-class patterns are all close to 0, demonstrating that all the patterns are correctly classified. The accuracy for this simple binary classification task is therefore 100%. After the pattern classification, the weights exhibit only slight changes (Fig. 5c), demonstrating good reliability of the FE-PS-NET as a pattern classifier (see Supplementary Fig. S15 and S16 for more discussion).

**Fig. 6:**
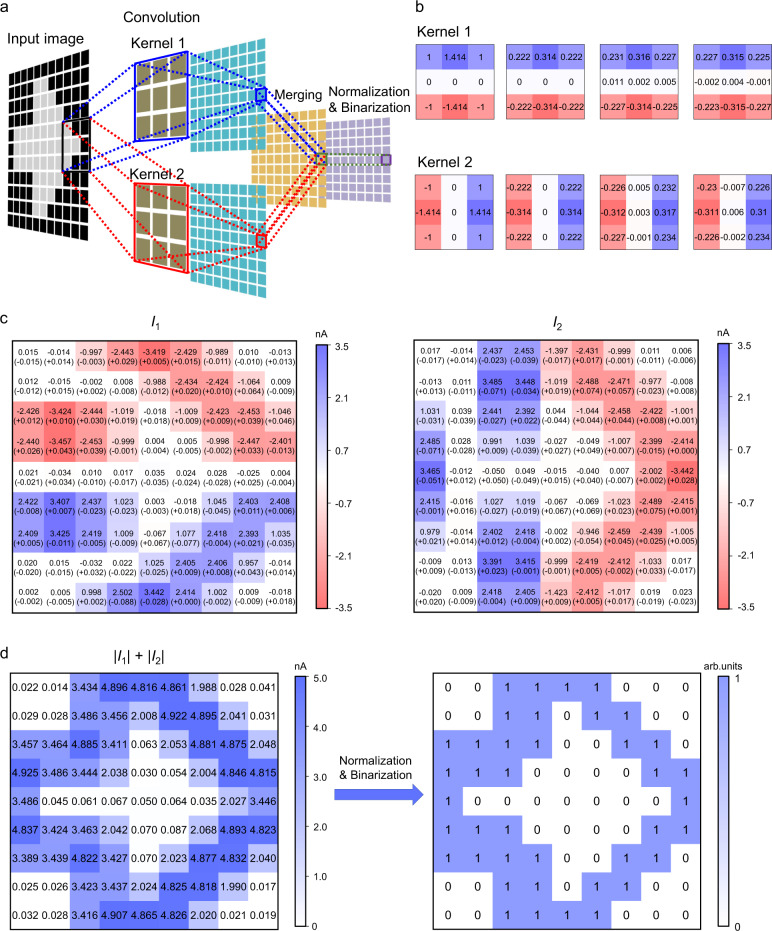
Implementation of edge detection. **a** Schematic illustration of the operations for the edge detection. The convolution is implemented by sequentially presenting the sub-images whose pixel values are translated to the illumination signals to the kernels based on FE-PS-NET. The merging, normalization, and binarization of output currents are performed in software. **b** From left to right: theoretical dimensionless weights, theoretical photoresonsivities scaled from dimensionless weights, actual weights right after programming, and actual photoresonsivities after edge detection, for Kernel 1 and 2. The unit of photoresponsivity is mA/W (not shown). **c** Output currents *I*_1_ (left panel) and *I*_2_ (right panel) after the convolution with the two kernels. The values outside the brackets are the actual *I*_1_ and *I*_2_ values while those in the brackets are the differences between the theoretical *I*_1_ and *I*_2_ values and their corresponding actual values. **d** Image obtained by merging *I*_1_ and *I*_2_ (left panel) and final output image after normalization and binarization showing the detected edge (right panel).

Another important image processing functionality, i.e., the edge detection, was also demonstrated. As shown in Fig. 6a, an 11 × 11 image showing an arrow sign was used as the input image. The pixel values in this image were binarized, in a way similar to that used for the pattern classification. For the convolution operation in the edge detection, 3 × 3 kernels were used to slide over the input image with a stride of 1. Consequently, the initial input image was decomposed into 81 3 × 3 sub-images. These sub-images, with pixel values translated to illumination signals, were presented sequentially to the kernels based on FE-PS-NET. The kernels used here were two Sobel kernels, as displayed in Fig. 6b. These two kernels were implemented with a 2 × 9 (*M* = 2 and *N* = 9) FE-PS-NET. The kernel weights were mapped to the photoresponsivities of the corresponding FE-PSs (Fig. 6b and Supplementary Note 2). During the convolution, the dot product between a sub-image and a kernel was obtained through the MAC process in the FE-PS-NET (see Fig. 6a). After the convolution, two sets of output current data *I*_1_ and *I*_2_, corresponding to Kernel 1 and 2, respectively, were collected. They were further merged, normalized, and binarized to form the output image (Methods).

Figure 6c shows the output currents *I*_1_ and *I*_2_ after the convolution with the two kernels. All the actual current values agree well with the theoretically calculated ones. Figure 6d (right panel) presents the final output image, clearly revealing the edge between the arrow and the background (note: the edge contains both the outermost pixels of foreground and innermost pixels of background). Based on Fig. 6d, a performance metric, i.e., F-Measure, is calculated to be 1 (Methods). This demonstrates the good performance of edge detection implemented with the FE-PS-NET. In addition, almost no changes are observed in the kernel weights after the edge detection (Fig. 6b), verifying the reliability of the FE-PS-NET as an edge detector (see Supplementary Fig. S17 for more discussion).

The high accuracy and reliability of FE-PS-NET for image processing can be well attributed to the reliable polarization control of photoresponsivity, as demonstrated previously. In addition, the low latency is another merit of FE-PS-NET because it can simultaneously sense and process images in the analog domain. The operation speed is thus limited mainly by the photocurrent generation time and the RC time constant of the circuit. Due to time resolution limit of our measurement system, we can only confirm that the photocurrent generation time is below 100 ms (Supplementary Fig. S18). Indeed, the photocurrent generation in an FE-PS can occur within 1 ns^24,25^; hence, the RC time constant of the circuit may become the major speed-limiting factor. A rough estimation shows that the total latency of sensing and processing a 10-million-pixel image is ~2.6 μs for the FE-PS-NET, which is 4 orders of magnitude shorter than that of a conventional Von Neumann system (Supplementary Note 3). In terms of energy consumption, because the FE-PS operates in the gate voltage-free photovoltaic (i.e., self-powered) mode, zero energy is in principle consumed when performing the inference. The energy is consumed only when programing the FE-PS. As shown in Supplementary Fig. S19, applying ±2 V/10 μs programming pulses to our ~0.0314 mm^2^ PZT-based FE-PS results in an average energy consumption of ~3.1 nJ. As the FE-PS could be scaled down to ~1 μm^2^^49^, the energy consumption may thus be reduced to ~0.1 pJ per bit per operation, which is a sufficiently low value compared with those of recent emerging neuromorphic devices^14,32^. The good scalability also allows the construction of a large-scale FE-PS-NET in a small area. The area efficiency could further benefit from the following two factors. First, the FE-PS-NET stores the weights locally, and thus no external memory is needed to remember the weights. In addition, a single FE-PS can represent both positive and negative weights, making it unnecessary to use a pair of FE-PSs to represent a signed weight. The above features including high speed, scalability, and reliability, as well as low energy consumption, make the FE-PS-NET a good candidate for the hardware implementation of real-time machine vision.

## Discussion

In summary, we achieved a proof-of-concept demonstration of FE-PS-NET that can simultaneously sense and process images. The FE-PS-NET was constructed by wiring multiple FE-PSs with each FE-PS consisting of a two-terminal Pt/PZT (epitaxial film)/SRO heterostructure. The FE-PS exhibited multilevel nonvolatile photoresponses as well controlled by the remanent polarization. Also benefitting from the polarization control, small cycle-to-cycle and device-to-device variations (~3% and ~3.2%, respectively), as well as high endurance (1 × 10^6^ cycles), were demonstrated for the FE-PS. Moreover, the switching of the polarization direction induced the reversal of the photocurrent direction, thus enabling a single FE-PS to represent both positive and negative weights. Using the FE-PS as a building block, the FE-PS-NET exhibited the capability to perform in-sensor MAC operations. The FE-PS-NET was further demonstrated with real-time image processing functionalities, including binary classification between ‘X’ and ‘T’ patterns with 100% accuracy and edge detection for an arrow sign with an F-Measure of 1 (under 365 nm UV light). Moreover, because of the polarization-controlled photovoltaic operation mode, ultrafast photocurrent generation process, and in-sensor computing architecture, the FE-PS-NET could achieve high reliability, ultralow latency, and zero energy consumption for inference. This study demonstrates the first type of ferroelectric neuromorphic device with the sensing-memory-computing integrated paradigm, opening up a new way for the development of reliable, high-speed, and low-power hardware for real-time machine vision.

## Methods

### Device fabrication

~40 nm SRO and ~120 nm PZT epitaxial thin films were successively grown on (001)-oriented STO single crystalline substrates by PLD using a KrF excimer laser (*λ* = 248 nm). An energy fluence of 0.9 J/cm^2^ and a repetition rate of 5 Hz were used for the depositions of both SRO and PZT films. The SRO films were first deposited at a substrate temperature of 680 °C under an oxygen pressure of 15 Pa. The PZT films were subsequently deposited under the same oxygen pressure, but the substrate temperature was lowered to 600 °C. After growth, the PZT/SRO films were cooled to room temperature at a rate of 10 °C/min under 1 atm oxygen pressure. The Pt top electrodes with ~10 nm in thickness were ex situ deposited on the films through a shadow mask (diameter: ~200 μm) by PLD at room temperature and under vacuum. The individual Pt/PZT/SRO FE-PSs were thus formed. To construct an FE-PS-NET, the individual FE-PSs were connected by wiring (Supplementary Fig. 1).

### Characterizations

The crystalline structure and phase purity of the films were investigated by XRD (‘X’ Pert PRO, PANalytical). The epitaxial quality and microstructure were further examined using TEM (Tecnai G2-F20). The surface morphology and domain structure were characterized by AFM and PFM, respectively, which were performed on an integrated scanning probe microscope (Asylum Research MFP-3D) with Pt-coated silicon tips (Nanoworld EFM Arrow). The PFM amplitude and phase images were acquired by using an AC driving voltage of 0.8 V in the DART (dual a.c. resonance tracking) mode.

### Electrical measurements

The bipolar and monopolar *P–V* hysteresis loops were measured with triangular pulses on a ferroelectric workstation (Radiant Precision Multiferroic). The *I–V* characteristics were measured with a SourceMeter (Keithley 6430). Both the ferroelectric workstation and SourceMeter were used to apply electrical pulses with various amplitudes and widths. In the photovoltaic measurement, 365 nm UV light-emitting diodes (LEDs) with tunable light intensities were used as the light sources while the SourceMeter recorded the photocurrent data. When applying illumination to an individual FE-PS, without silver paste on the top electrode the whole electrode area was considered for the calculation of optical power. However, for the FE-PS in FE-PS-NET, only the area without the coverage of the silver paste was considered as being subjected to the illumination and used for the calculation of optical power.

### Simulations

For the pattern classification task, the sigmoid activation function which was implemented in software is expressed as:

3 $$f(x)=\frac{1}{1+{e}^{-x}},$$

4 $$x=\alpha {I}_{1},$$

where *x* is the neuronal input scaled from the measured current *I*_1_, and *α* is a scaling factor (*α* = 3 nA^−1^ in this work).

For the edge detection task, two Sobel kernels were used for the convolution, which are expressed as:

5 $${{{{{\rm{Kernel}}}}}}\,1=\left[\begin{array}{ccc}1 & \sqrt{2} & 1\\ 0 & 0 & 0\\ -1 & -\sqrt{2} & -1\end{array}\right],$$

6 $${{{{{\rm{Kernel}}}}}}\,2=\left[\begin{array}{ccc}-1 & 0 & 1\\ -\sqrt{2} & 0 & \sqrt{2}\\ -1 & 0 & 1\end{array}\right].$$

After the convolution, two maps of output current data (*I*_1_ and *I*_2_, corresponding to Kernel 1 and 2, respectively) were obtained. The two maps were merged into one map following the equation below:

7 $${I}_{{{{{{\rm{E}}}}}}}=|{I}_{1}|+|{I}_{2}|.$$

Then, the *I*_E_ values were normalized to the range [0, 1]. The normalized *I*_E_ values were further binarized as follows:

8 $${{{{{\rm{Normalized}}}}}}\,{I}_{E}=\left\{\begin{array}{c}0,x\, < \, d\\ 1,x\ge d\end{array},\right.$$

where *d* is a threshold value, and *d* = 0.6 was used in this work.

After the normalization and binarization, the final output image showing the detected edge was obtained. The F-Measure was used to evaluate the quality of the output image, as given by

9 $${{{{{\rm{F}}}}}}-{{{{{\rm{Measure}}}}}}=(1+{\beta }^{2})\cdot \frac{precision\cdot recall}{{\beta }^{2}\cdot precision+recall},$$

where *β* is a constant (*β* = 1 was used here). The *precision* and *recall* are expressed as

10 $$precision=\frac{TP}{TP+FP},$$

11 $$recall=\frac{TP}{TP+FN},$$

where *TP*, *FP*, and *FN* are the numbers of true positives, false positives, and false negatives, respectively. The detected edge shown in Fig. 6d is exactly the actual edge; in other words, all the 40 edge pixels (blue color in Fig. 6d) are correctly detected and none of the non-edge pixels (white color in Fig. 6d) are wrongly detected as edge pixels. Therefore, the values of *TP*, *FP*, and *FN* are 40, 0, and 0, respectively. According to Eqs. (9–11), the values of *precision*, *recall*, and F-measure are all calculated to be 1.

### Reporting summary

Further information on research design is available in the Nature Research Reporting Summary linked to this article.

## Supplementary information

Supplementary Information

Reporting Summary

Lasing Reporting Summary

## Data Availability

The data that support the findings of this study are available from the corresponding author upon reasonable request.
