# Supplementary material for: Ferroelectric photosensor network: an advanced hardware solution to real-time machine vision
Source: Nat Commun. 2022 Mar 31;13:1707. doi: 10.1038/s41467-022-29364-8 (PMC8971381; doi:10.1038/s41467-022-29364-8)
Supplement: Supplementary file 1 — Supplementary Information [file 41467_2022_29364_MOESM1_ESM.pdf]

Supplementary Information

Ferroelectric photosensor network: an advanced hardware solution to real-time machine vision

Boyuan Cui[1], Zhen Fan[1]*, Wenjie Li[1], Yihong Chen[1], Shuai Dong[1], Zhengwei Tan[1], Shengliang Cheng[1], Bobo Tian[2], Ruiqiang Tao[1], Guo Tian[1], Deyang Chen[1], Zhipeng Hou[1], Minghui Qin[1], Min Zeng[1], Xubing Lu[1], Guofu Zhou[3], Xingsen Gao[1], and Jun-Ming Liu[4]

[1] *Institute for Advanced Materials and Guangdong Provincial Key Laboratory of Optical Information Materials and Technology, South China Academy of Advanced Optoelectronics, South China Normal University, Guangzhou 510006, China*

[2] *Key Laboratory of Polar Materials and Devices, Ministry of Education, East China Normal University, Shanghai 200241, China*

[3] *National Center for International Research on Green Optoelectronics, South China Normal University, Guangzhou 510006, China*

[4] *Laboratory of Solid State Microstructures and Innovation Center of Advanced Microstructures, Nanjing University, Nanjing 210093, China*

*Email: fanzhen@m.scnu.edu.cn

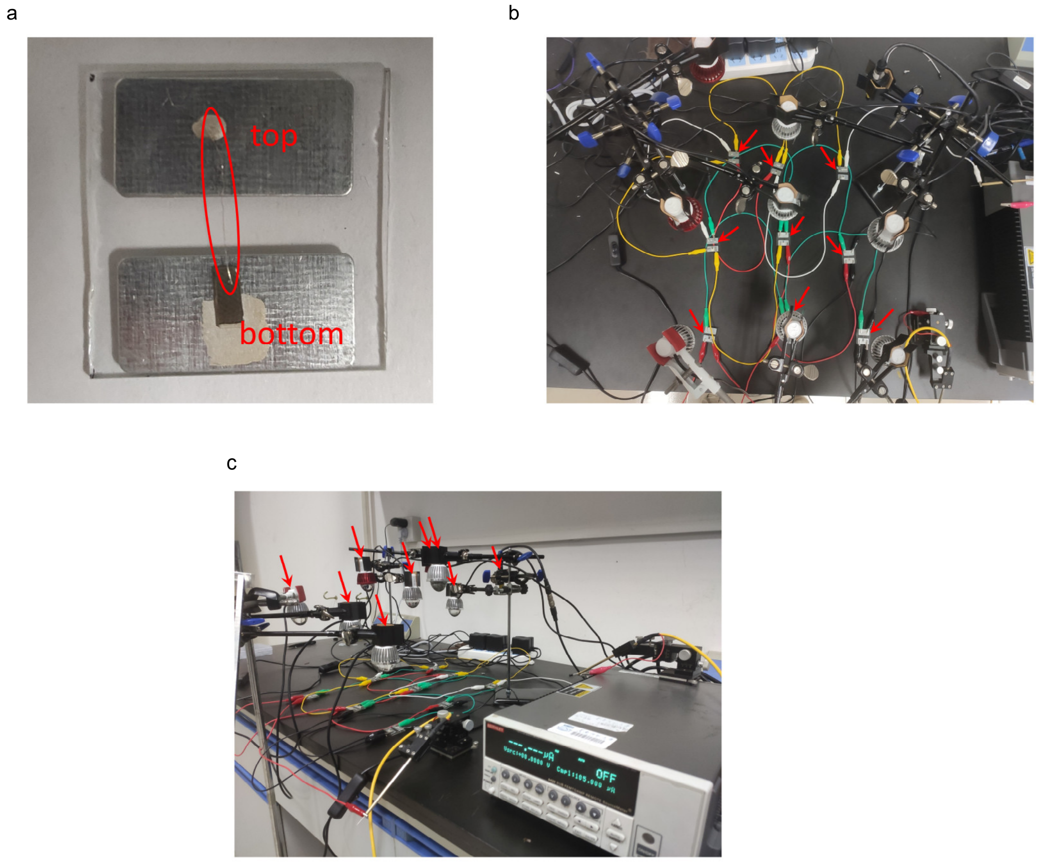

Figure S1. Photos of (a) a single FE-PS with the device structure of Pt/PZT/SRO, (b) a 1 × 9 FE-PS-NET, where the 9 FE-PSs are indicated by the red arrows, and (c) the experimental setup with the LEDs (indicated by red arrows) and the SourceMeter.

In Figure S1a, the Pt top electrode (diameter: ~200 μm) is connected to the upper iron plate via a Pt wire (diameter: 50 μm) whose two ends are pasted with silver paste, while the bottom SRO electrode is connected to the lower iron plate via the silver paste. The two iron plates can now act as the top and bottom electrodes of this FE-PS, and they can be conveniently connected to other FE-PSs by using the wires with alligator clips (see Figure S1b).

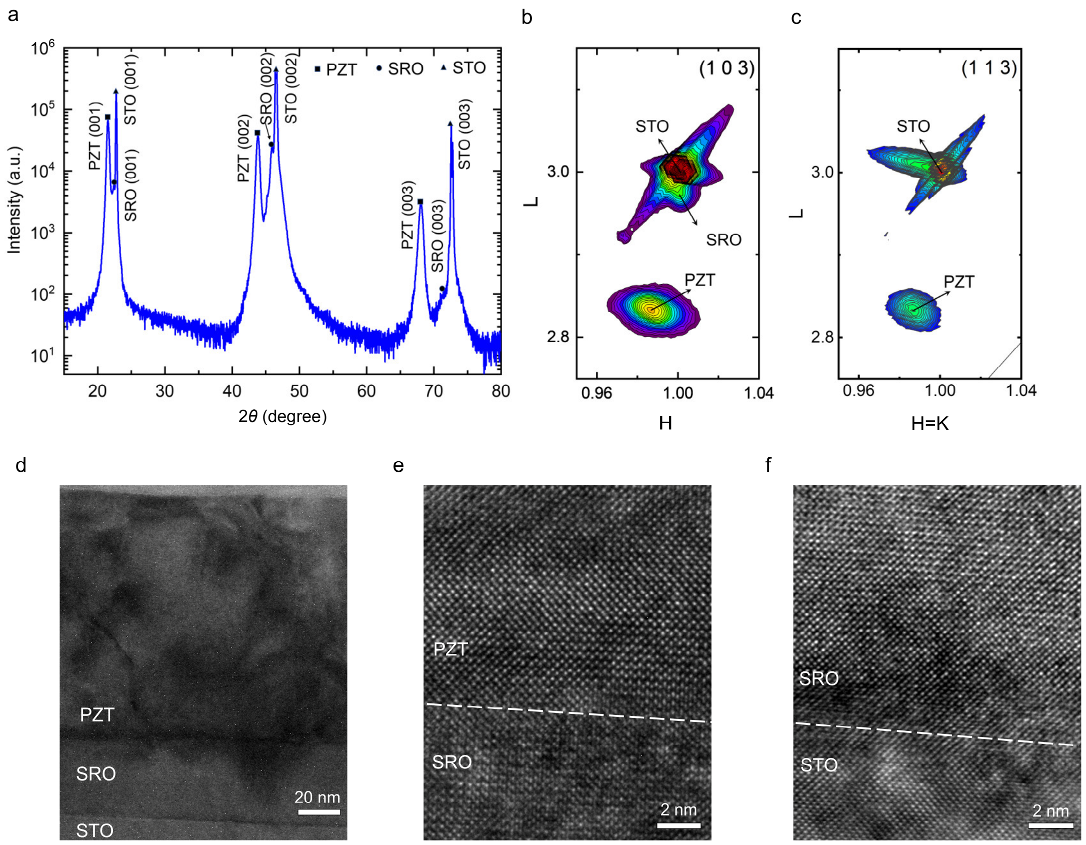

Figure S2. (a) XRD θ-2θ diffraction pattern, RSMs around STO (b) (103) and (c) (113) reflections, (d) low-magnification TEM image, and high-magnification TEM images around (e) PZT/SRO and (f) SRO/STO interfaces.

As shown in Figure S2a, only the (00*l*) peaks from PZT, SRO and STO are present, confirming the phase purity of the PZT and SRO films (containing only the perovskite phases). Figure S2b,c show the reciprocal space mappings (RSMs) around the STO (103) and (113) reflections, respectively. Both the (103) and (113) reflections from PZT give rise to single diffraction spots, indicating that the PZT film is epitaxially grown on the SRO-buffered STO substrate with an in-plane orientational relationship of PZT [100] // STO [100]. Note that no diffraction spot from SRO is observed in Figure S2c, because it is not allowed for the P*nma* symmetry of SRO.[1]

Figure S2d shows that the PZT/SRO/STO heterostructure is well established and both PZT/SRO and SRO/STO interfaces are quite sharp. The thicknesses of the PZT and SRO

layers are determined as ~120 and ~40 nm, respectively. Some dark lines are observed in the PZT layer, which may be attributed to the domain walls or misfit strain-induced dislocations.[2,3] Figure S2e,f present the high-magnification TEM images taken around the PZT/SRO and SRO/STO interfaces, respectively. Well-aligned PZT and SRO lattices are observed, further confirming the epitaxial growth of both PZT and SRO layers.

Combing the XRD, RSM, and TEM results (Figure S2 and 2c in the main text), the out-of-plane and in-plane lattice constants of PZT are determined as 4.135 ± 0.005 Å and 3.945 ± 0.010 Å, respectively. These lattice parameters of PZT agree with those reported previously for the PZT films grown on the STO substrates.[4]

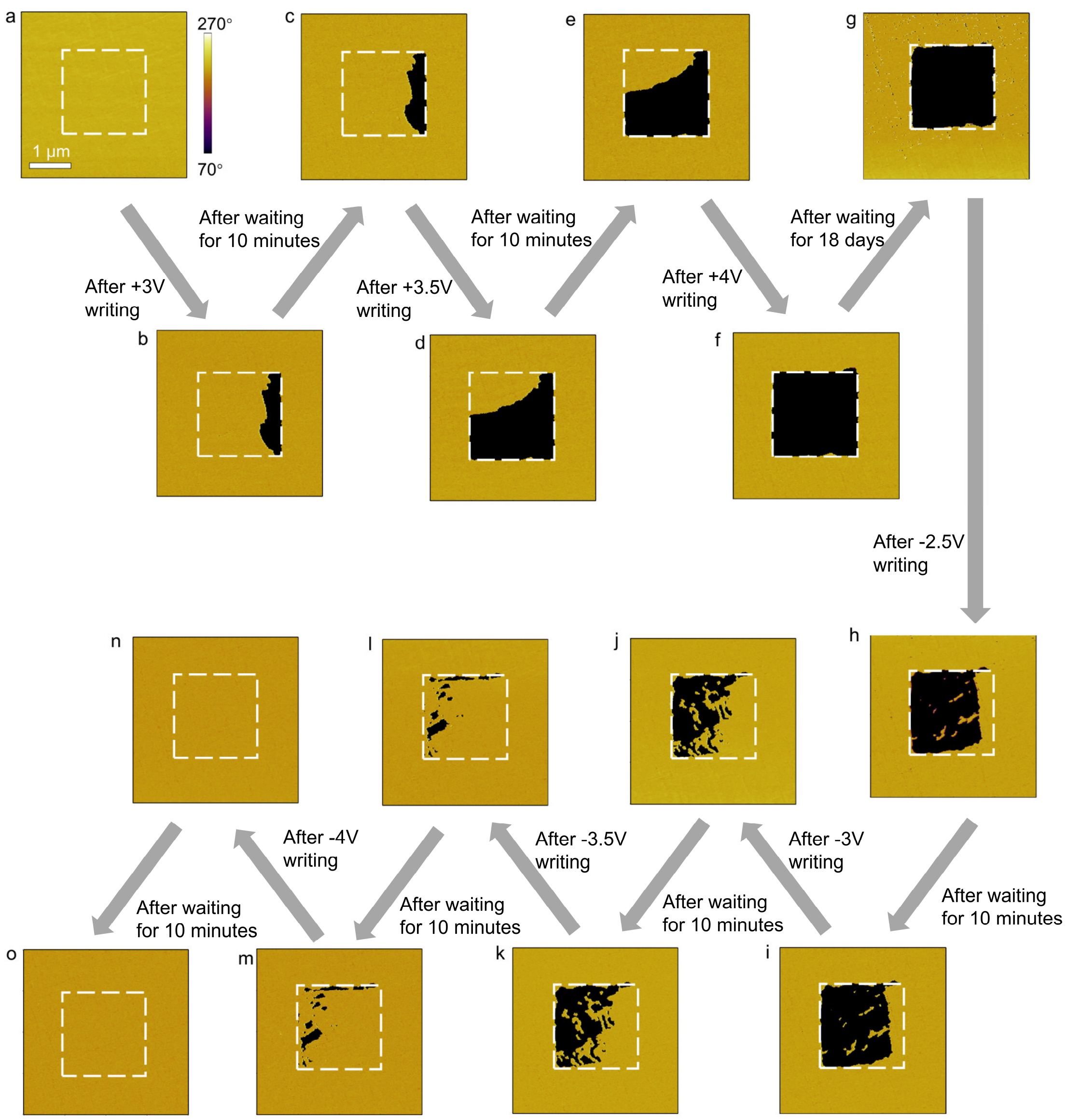

Figure S3. (a) Initial out-of-plane PFM phase image, and those measured (b,d,f,h,j,l,n) immediately after and (c,e,g,i,k,m,o) a few moments later after writing a 2 × 2 box sequentially with tip biases of (b,c) +3V, (d,e) +3.5V, (f,g) +4V, (h,i) -2.5V, (j,k) -3V, (l,m) -3.5V, (n,o) -4V. The waiting times before the PFM image scans are indicated in the corresponding panels.

It is seen that applying positive (negative) write voltage results in the black (yellow) contrast in the PFM phase image. Therefore, the black and yellow contrasts correspond to the downward and upward domains, respectively. As the positive write voltage increases

(Figure S3b-g), a downward domain (in the right area) emerges, expands, and eventually fills up the whole written area. As the negative write voltage increases (Figure S3h-o), a major upward domain (in the right area) and some minor upward domains (in the middle area) are formed and they gradually expand to dominate the whole written area. The upward/downward mixed domain configurations are observed during the domain switching process, well accounting for the formation of multiple intermediate polarization states. Moreover, Figure S3c, e, g, i, k, m, and o show that all the domain states are stable. In particular, the retention of the +4 V written state was tested for 18 days. Almost no contrast changes are observed in the written area, confirming the stability of the switched domains.

To understand the origin of good domain stability, the observed domain switching mode may be further analyzed. As indicated by Figure S3, the domain switching is mainly contributed by the growth of a major switched domain. This suggests that the switched domain already penetrates through the film thickness and grows sideways. As a result, the switched domain can be well screened by charges in the two electrodes (or charges from the ambient if there is no top electrode), thus reducing the depolarization effect and enhancing the domain stability. In addition, the domain connecting the two electrodes can facilitate the transport of photo-excited charge carriers within the domain, which may contribute to the photocurrent stability.

Then, let's turn to the observation that the switched domain first emerges in the right part of the written area (Figure S3b,h), which may be explained as follows. Ferroelectric films generally contain nucleation centers with spatial and energy distributions. In the specific written area as shown in Figure S3, the right region may contain nucleation centers with relatively low activation energies. Therefore, when a critical field is applied, the reverse domains are first nucleated and grown in the right region. However, in other written areas, where the reverse domains first emerge can be different (results not shown). This may be due to that the distributions of nucleation centers with relatively low activation energies are different in different selected areas.

Also note that the coercive voltages revealed by PFM (Figure S3b,h) and *P-V* measurement (Figure 2d in the main text) are quite different. In the PFM, the AFM tip is

used as the top electrode; therefore, the electric field distribution is different from that in the *P-V* measurement. In addition, there exists a contact resistance between the AFM tip and the sample surface, causing the voltage drop at the tip/sample interface. These two major factors may lead to the different coercive voltages observed in the PFM and *P-V* measurement.

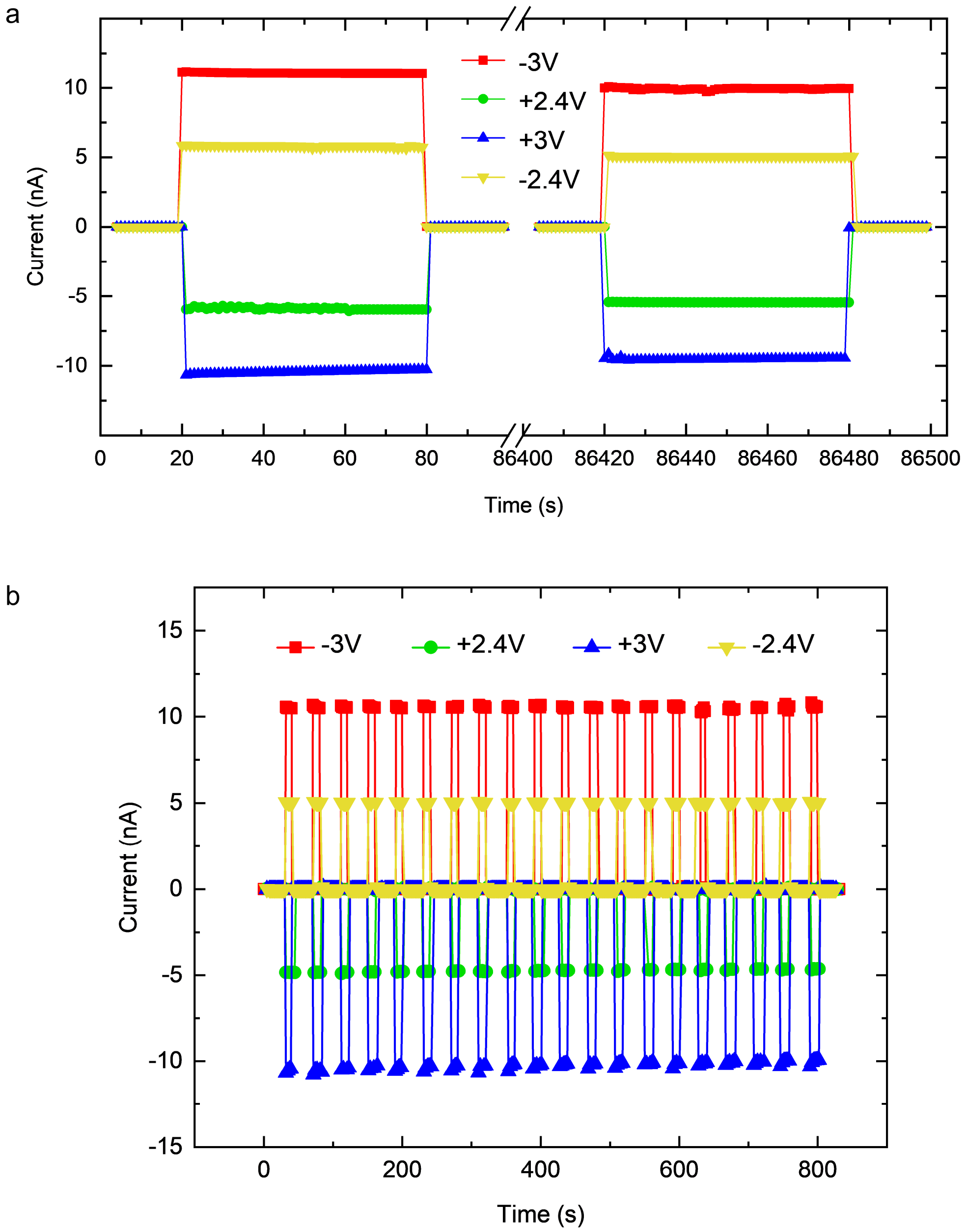

Figure S4. (a) Long-term stability and (b) cyclability of photocurrents of the Pt/PZT/SRO FE-PS in different polarization states as set by applying -3 V, +2.4 V, +3 V, and -2.4 V pulses (pulse width: 0.15 ms) sequentially. In b, the light is switched ON and OFF alternately with 10 and 30 seconds for the ON and OFF periods, respectively.

Four different polarization states: complete P_{up}, incomplete P_{down}, complete P_{down}, and incomplete P_{up} states, were obtained by sequentially applying -3 V, +2.4 V, +3 V, and -2.4

V pulses, respectively. The light intensity was ~150 mW/cm^2 and the device area was ~0.0314 mm^2 (not covered by the silver paste). Figure S4a shows that the photocurrents measured in all the polarization states are quite stable. Such different photoresponsive states can be retained for at least 24 hours. Figure S4b demonstrates that the photoresponsive states are reproducible during the frequent ON/OFF illumination cycling.

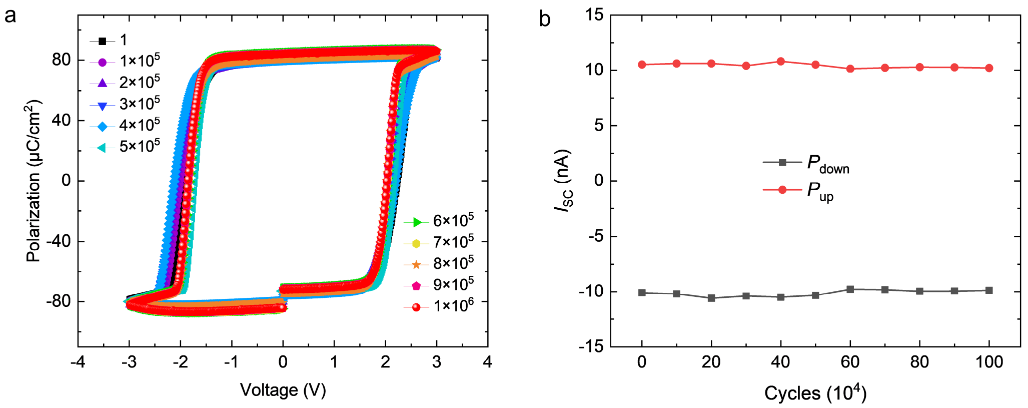

Figure S5. (a) *P*-*V* hysteresis loops and (b) photocurrents in the complete P_{up} and P_{down} states measured for the Pt/PZT/SRO FE-PS after different fatigue cycles.

The FE-PS was switched by 3 V/10 μs pulses for 10^6 cycles. As shown in Figure S5, both the *P*-*V* hysteresis loops and the photocurrents (light intensity: ~150 mW/cm^2; device area: ~0.0314 mm^2) show almost no changes during the fatigue test, demonstrating the good endurance of the FE-PS.

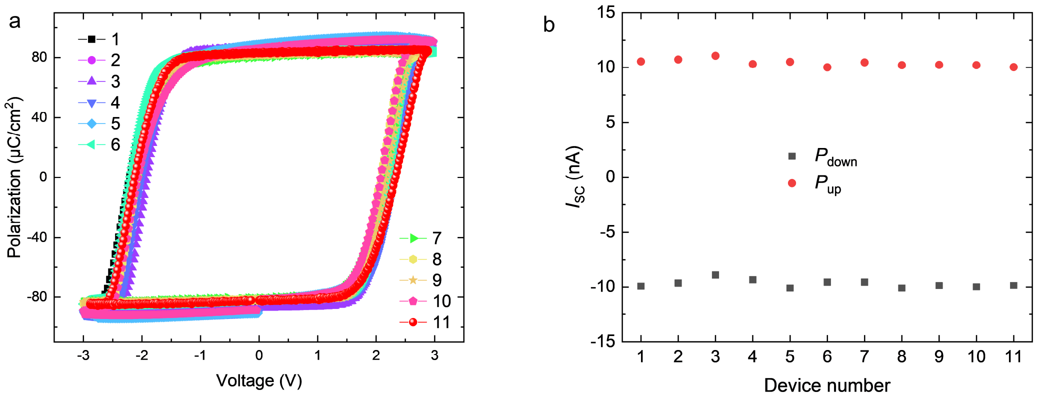

Figure S6. (a) *P*-*V* hysteresis loops and (b) photocurrents in the complete P_{up} and P_{down} states measured for 11 Pt/PZT/SRO FE-PSs.

The different FE-PSs exhibit similar polarization switching behavior and photocurrent responses (light intensity: ~150 mW/cm^2; device area: ~0.0314 mm^2). The device-to-device variation of photocurrents is estimated to be ~3.2% for the FE-PSs.

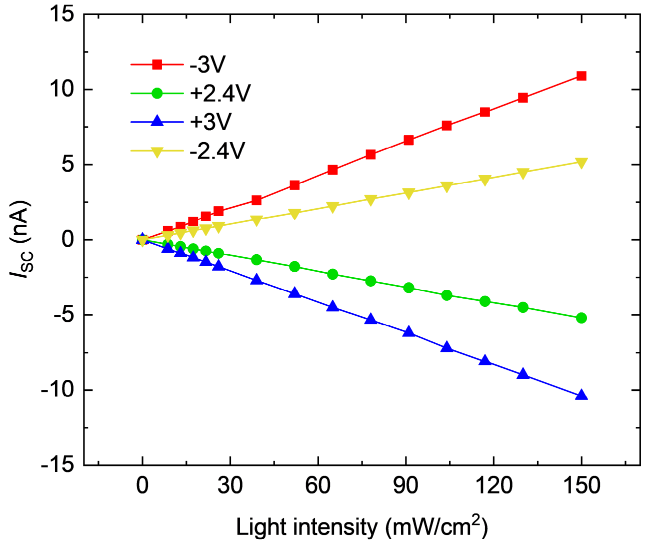

Figure S7. Dependences of photocurrent on light intensity for the Pt/PZT/SRO FE-PS (device area: ~0.0314 mm^2) in different polarization states as set by applying -3 V, +2.4 V, +3 V, and -2.4 V pulses sequentially.

The photocurrents in all the different polarization states exhibit linear relationships with the light intensity. The linearities of these curves are greater than ~0.94. The linear dependence of photocurrent on light intensity allows the photoresponsivity to be well defined. It is also a prerequisite for the multiplication operation in the FE-PS.

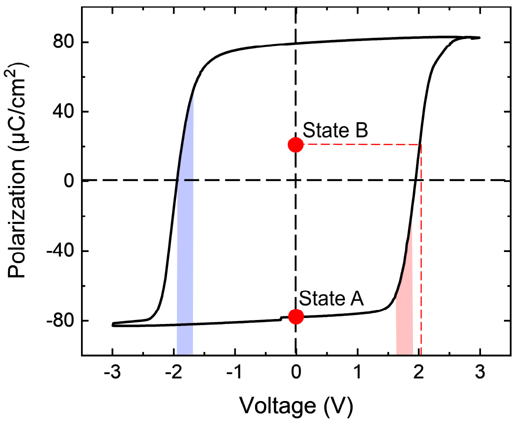

Figure S8. *P*-V loop of the specific device used for the LTP/LTD measurement. The colored regions indicate the ranges of applied pulse voltages for the LTP/LTD measurement.

The coercive voltages of the device used for the LTP/LTD measurement are ~±1.9 V, which are slightly lower than those of the device shown in Figure 2d in the main text. The colored regions in Figure S8 indicate the ranges of applied pulse voltages, i.e., from 1.65 V to 1.89 V in increments of 0.01 V for LTD and from -1.7 V to -1.94 V in decrements of 0.01 V for LTP (pulse shape: triangular; pulse width: 10 μs). Note that the colored regions are used only for the eye guide; however, the polarizations switched by the applied pulses may not be those shown in the colored regions because the applied pulses have the width of 10 μs, which is different from that of the pulse used for the *P-V* loop measurement (i.e., 0.15 ms).

The pulse voltages were purposely set to be lower than the coercive voltages while increased in magnitude, because we intended to switch the polarization gradually to produce many intermediate states. Although each pulse could switch only a small amount of polarization, the accumulative effect of all the pulses could lead to the complete switching between P_{up} and P_{down} (to be evidenced in Figure S9-S12). However, if a pulse with amplitude larger than the coercive voltage is applied, the polarization state of the device may be switched from State A directly to State B, making the intermediate states between State A and State B unavailable (see Figure S8).

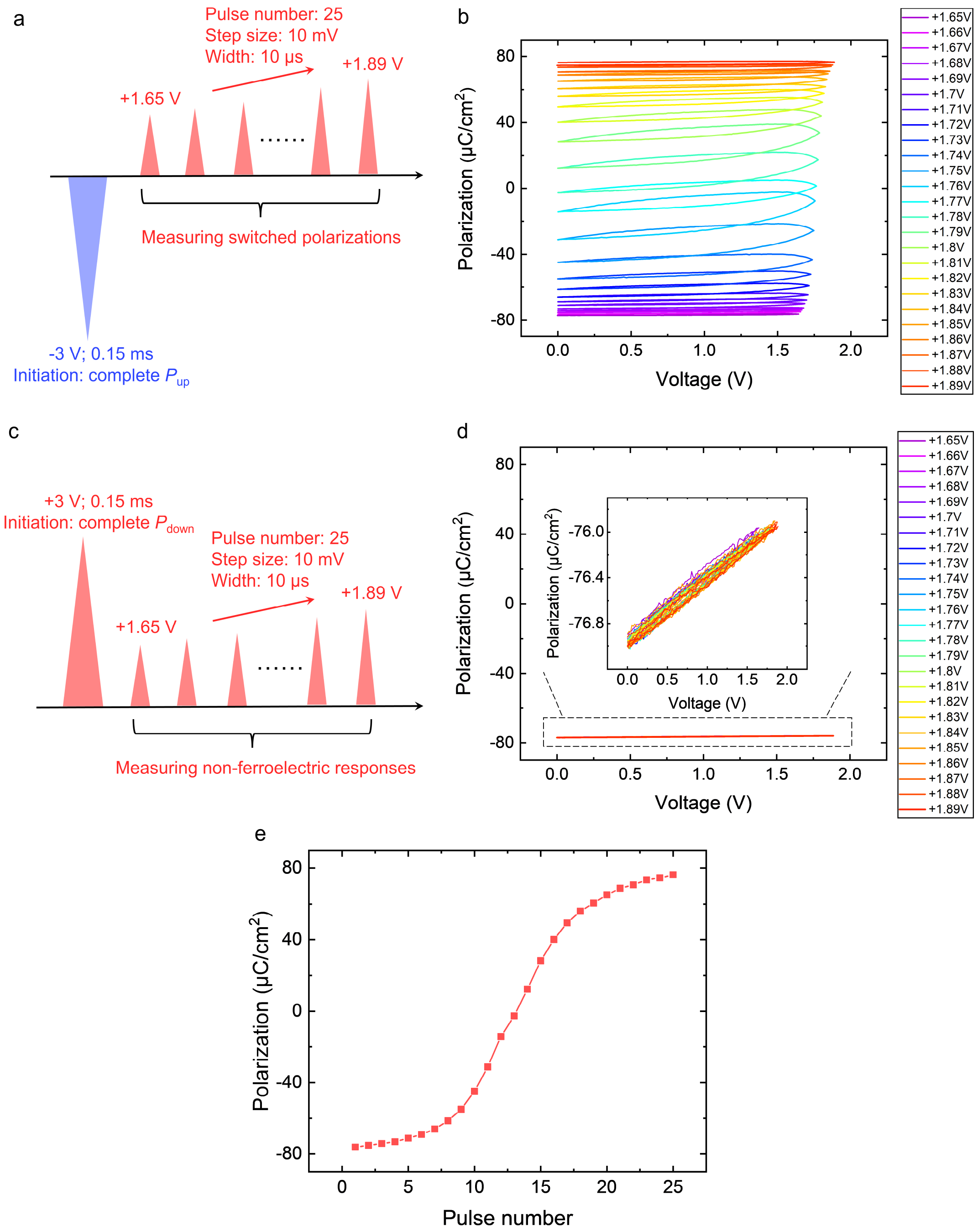

Figure S9. (a) Schematics of the positive pulses from 1.65 V to 1.89 V applied after a -3 V preset pulse and (b) *P*-*V* loops measured by these pulses showing the switched polarizations. (c) Schematics of the positive pulses from 1.65 V to 1.89 V applied after a +3 V preset pulse and (d) *P*-*V* loops measured by these pulses showing the non-ferroelectric contributions with inset showing the enlarged view. The -3 V and +3 V preset pulses are

used to initialize the device into the complete P_{up} and P_{down} states, respectively. (e) Polarization evolution as a function of pulse number during the application of positive pulses from 1.65 V to 1.89 V.

To demonstrate that the pulses applied in the LTP/LTD measurements can switch the polarization, we measured the *P*-*V* loops induced by these pulses (method illustrated in Figure S9a). Figure S9b shows the *P*-*V* loops obtained by applying the positive pulses from 1.65 V to 1.89 V to the device with an initial complete P_{up} state (preset by a -3 V/0.15 ms pulse). These loops show hysteresis windows with different window sizes, implying that the polarizations (albeit with different magnitudes) can be switched by these pulses. To exclude the possibility that the hysteresis windows are caused by non-ferroelectric responses (e.g., leakage current), we also measured the *P*-*V* loops by applying the same positive pulses to the device with an initial complete P_{down} state (method illustrated in Figure S9c). Because the device was already in the complete P_{down} state, the positive pulses would induce only non-ferroelectric responses rather than polarization switching. The resultant loops are shown in Figure S9d, all of which exhibit very small hysteresis windows with negligible "nominal" polarization changes, indicating that non-ferroelectric responses are very small. Therefore, it is confirmed that the positive pulses applied in the LTD measurement can switch the polarization.

Moreover, because these pulses were applied successively (no preset pulses were inserted between them), the switched polarizations can thus be accumulated to obtain the polarization state after each pulse. Figure S9e shows the polarization evolution as a function of pulse number during the application of the positive pulses from 1.65 V to 1.89 V. One can see that the polarization gradually changes from ~-80 μC/cm^2 to ~+80 μC/cm^2, demonstrating that the gradual switching from the complete P_{up} state to the complete P_{down} state is realized by applying these positive pulses.

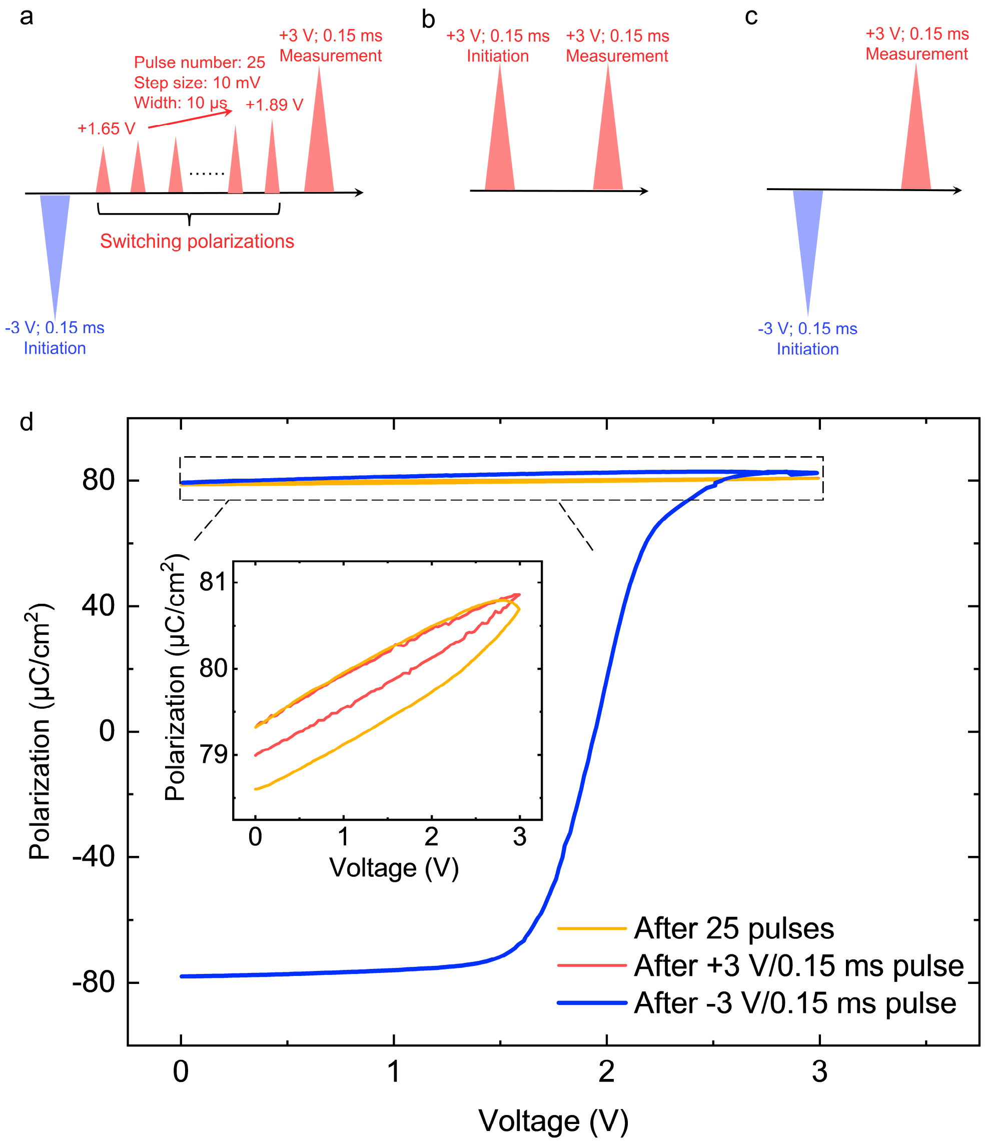

Figure S10. Schematics of the polarization measurements (measurement pulses: +3 V) conducted after applying (a) 25 pulses from 1.65 V to 1.89 V, (b) +3 V preset pulse, and (c) -3 V preset pulse. (d) Comparison of the *P*-*V* loops measured in the different states as described in a-c, with the inset showing the enlarged view. Applying -3 V and +3 V preset pulses can initialize the device into the complete P_{up} and P_{down} states, respectively.

To further confirm that the complete P_{down} state can eventually be reached after applying the 25 pulses from 1.65 V to 1.89 V, a +3 V/0.15 ms pulse was applied to check the final polarization state (method illustrated in Figure S10a-c). As shown in Figure S10d,

the P-V loop measured after applying pulses from 1.65 V to 1.89 V exhibits a very small hysteresis window, which is similar to that measured in the complete P_{down} state but significantly different from that measured in the complete P_{up} state. It is therefore confirmed that the final polarization state after applying the pulses from 1.65 V to 1.89 V is almost the complete P_{down} state.

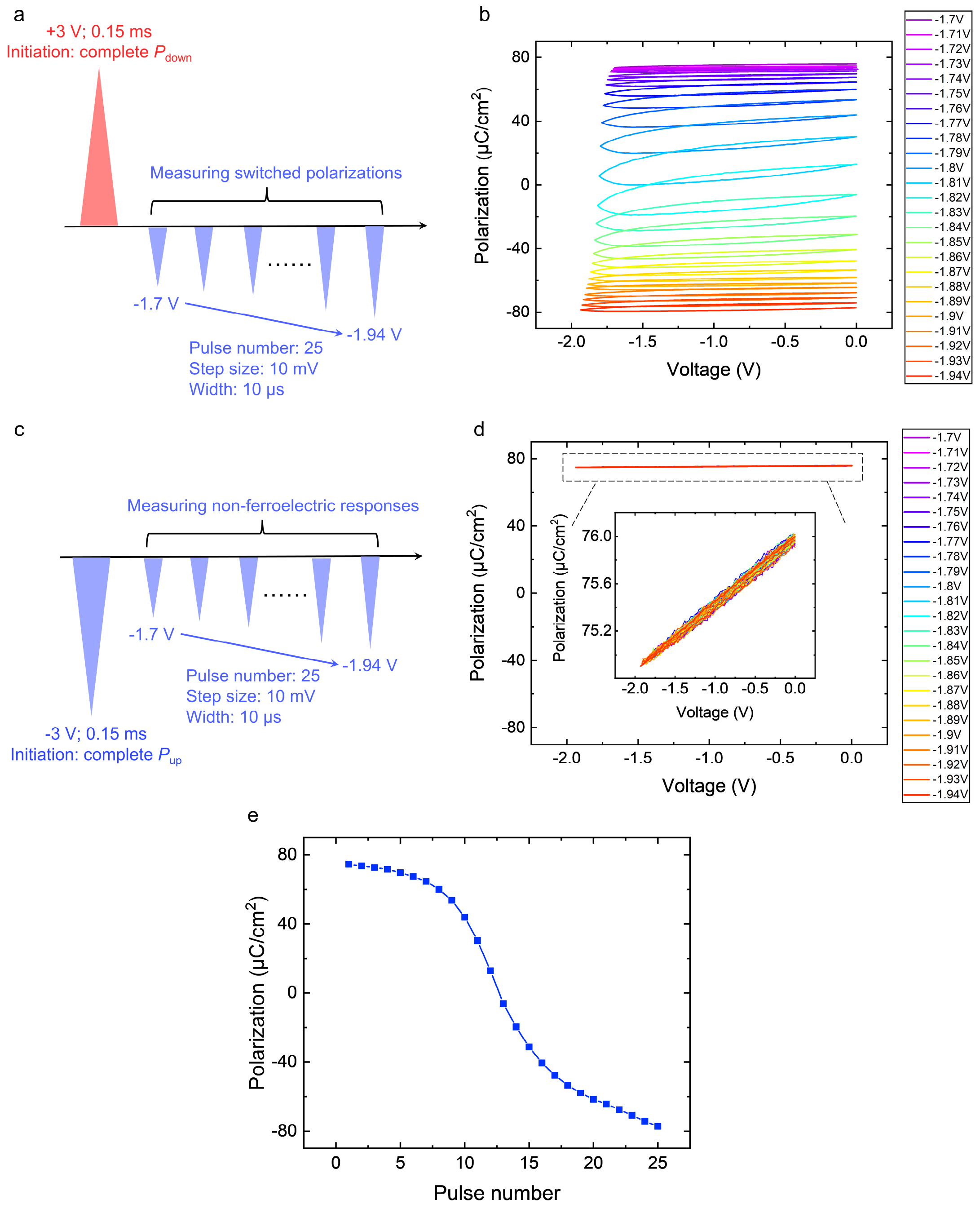

Figure S11. (a) Schematics of the negative pulses from -1.7 V to -1.94 V applied after a +3 V preset pulse and (b) *P-V* loops measured by these pulses showing the switched polarizations. (c) Schematics of the negative pulses from -1.7 V to -1.94 V applied after a -3 V preset pulse and (d) *P-V* loops measured by these pulses showing the non-ferroelectric contributions with inset showing the enlarged view. The -3 V and +3 V preset pulses are

used to initialize the device into the complete P_{up} and P_{down} states, respectively. (e) Polarization evolution as a function of pulse number during the application of negative pulses from -1.7 V to -1.94 V.

Similar to Figure S9 and S10, Figure S11 and S12 demonstrate that applying negative pulses from -1.7 V to -1.94 V can gradually switch the polarization state from P_{down} to P_{up}.

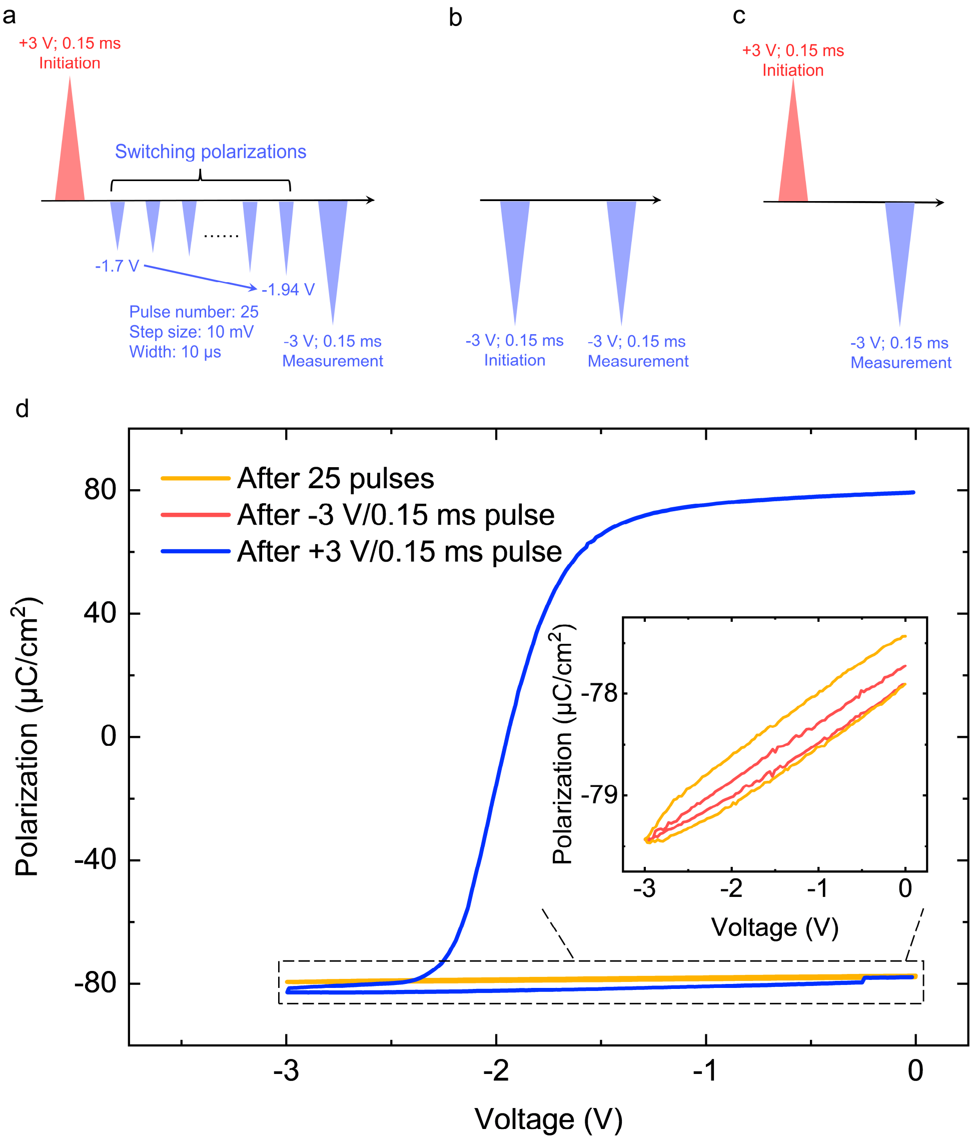

Figure S12. Schematics of the polarization measurements (measurement pulses: -3 V) conducted after applying (a) 25 pulses from -1.7 V to -1.94 V, (b) -3 V preset pulse, and (c) +3 V preset pulse. (d) Comparison of the *P*-*V* loops measured in the different states as described in a-c, with the inset showing the enlarged view. Applying -3 V and +3 V preset pulses can initialize the device into the complete P_{up} and P_{down} states, respectively.

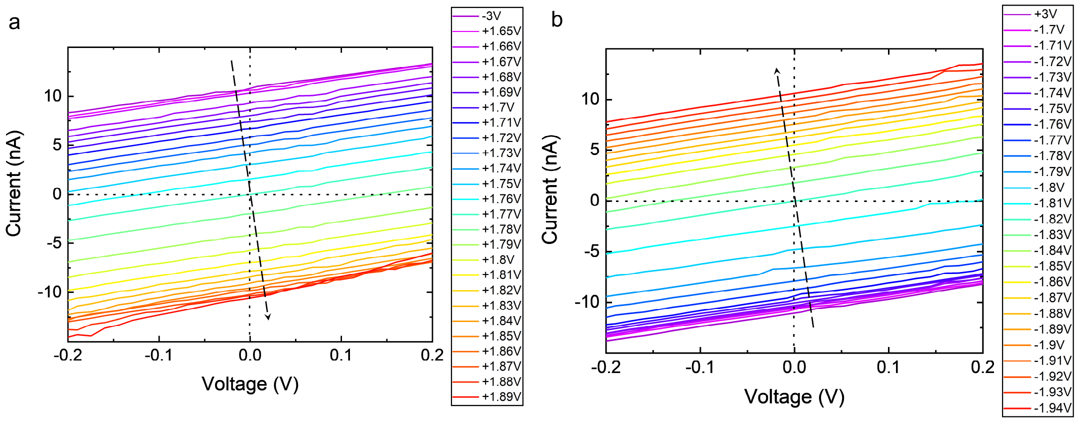

Figure S13. *I-V* curves under UV illumination after each of the pulses (a) from 1.65 V to 1.89 V and (b) from -1.7 V to -1.94 V.

We measured the *I-V* curves under UV illumination for the FE-PS (light intensity: ~150 mW/cm^2; device area: ~0.0314 mm^2) after each of the pulses from 1.65 V to 1.89 V and from -1.7 V to -1.94 V. These pulses were applied successively without preset pulses inserted between them. Before applying them, a -3 V/0.15 ms (or +3 V/0.15 ms) preset pulse was applied to initialize the device into the complete P_{up} (or P_{down}) state (note: the pulse scheme was the same as that used in Figure 3a in the main text). Figure S13a shows that the illuminated *I-V* curve shifts systematically along the positive voltage and negative current axes with the application of pulses from 1.65 V to 1.89 V, whereas the opposite shift is observed during the application of pulses from -1.7 V to -1.94 V (Figure S13b). It is thus revealed that the photocurrent (or photoresponsivity) evolves from positive maximum to negative maximum and back to positive maximum with the application of successive positive and negative pulses, well consistent with the results shown in Figure 3a in the main text.

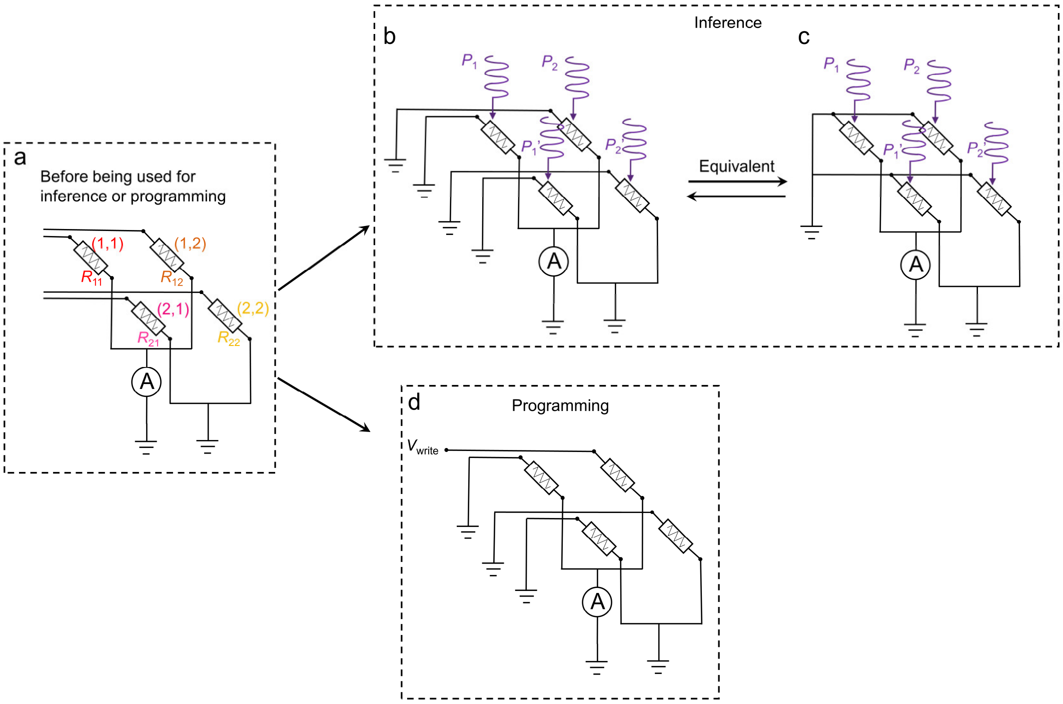

Figure S14. Schematic circuit diagrams of a 2 × 2 FE-PS-NET (a) before being used for inference or programming, (b) for inference and (c) its equivalent circuit, and (d) for programming.

Figure 4 in the main text shows only the schematic circuits of FE-PS-NET used for inference; however, the circuit for programming is not described. Here, a 2 × 2 ($M = 2$ and $N = 2$) FE-PS-NET is taken as an example to illustrate how the circuits are constructed in the two different cases: inference and programming. The circuit before being used for inference or programming is schematically shown in Figure S14a. When performing the inference, the wires connected to the upper ends of the four FE-PSs are grounded (see Figure S14b), and the equivalent circuit is schematically shown in Figure S14c (i.e., Figure 4f in the main text). When performing the programing to a specific FE-PS, e.g., the (1, 2) FE-PS, the wire connected to its upper end is subjected to the write pulse, while the wires connected to the upper ends of other FE-PSs are grounded (Figure S14d). In such case, no sneak path issue occurs in the circuit for programming.

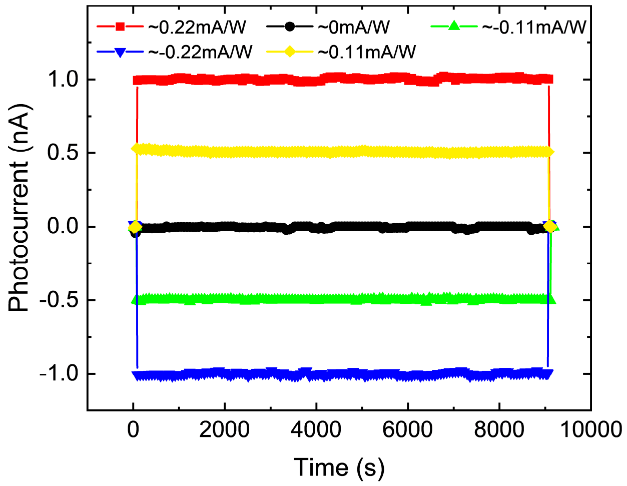

Figure S15. Time-dependent photocurrents in different photoresponsive states during a long-term UV illumination (optical power: ~4.5 μW).

As shown in Figure 5c and 6b in the main text, there are small changes of photoresponsivities after the experiments of pattern classification and edge detection. Note that during these experiments, the FE-PS was indeed not always under illumination (depending on the input pattern). To understand whether the photoresponsivity would exhibit a larger change if the FE-PS was continuously illuminated for a long time, we performed a long-term illumination for the FE-PS and monitored the time-dependent photocurrent. The illumination time was chosen to be 2.5 h, which was longer than the total experimental time of either pattern classification or edge detection. The optical power was ~4.5 μW.

As shown in Figure S15, the photocurrents in different photoresponsive states are quite stable within 2.5 h. Such good stability of photocurrent (or photoresponsivity) is attributed to the non-volatility of polarization. Based on Figure S15, the maximum relative change of photoresponsivity (σ_R) is estimated to be ≤5%.

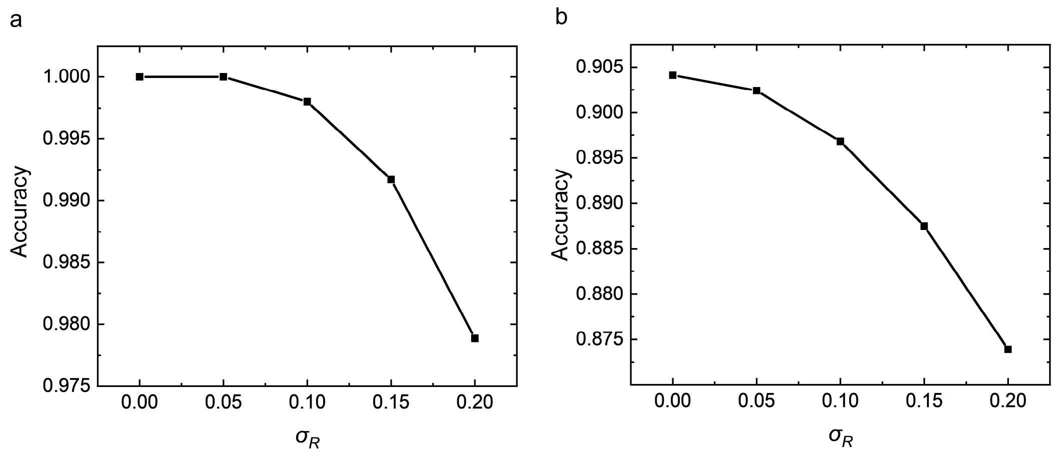

Figure S16. Simulated accuracies of (a) binary pattern classification and (b) MNIST image classification based on FE-PS-NET as a function of σ_R.

We studied the influence of σ_R on the performance of pattern classification based on FE-PS-NET by performing simulations. Single-layer perceptrons were used for simulation. The weights were added with randomly generated noises obeying a Gaussian distribution with a relative standard deviation of σ_R. At each σ_R, 10 sets of weights were generated and the test accuracies were averaged. Figure S16a shows the accuracy of binary pattern classification as a function of σ_R. It is seen that even when σ_R increases to 5%, the accuracy is still 100%. For a more complex task like MNIST image classification (Figure S16b), the accuracy loss caused by σ_R is within 0.2% when σ_R is below 5%. Because the experimentally observed σ_R is within 5% (Figure S15), its influence on the performance of the FE-PS-NET-based pattern classification is thus negligible.

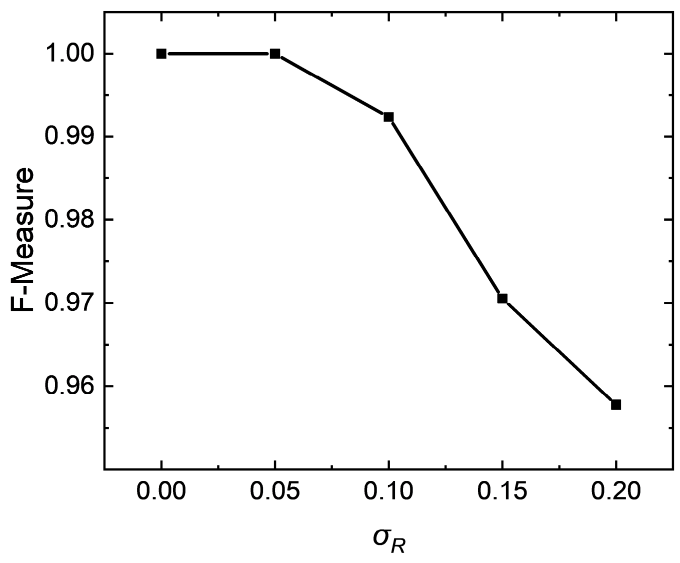

Figure S17. Simulated F-Measure of edge detection based on FE-PS-NET as a function of σ_R.

We studied the influence of σ_R on the performance of edge detection based on FE-PS-NET by performing simulations. The weights were added with randomly generated noises obeying a Gaussian distribution with a relative standard deviation of σ_R. At each σ_R, 10 sets of weights were generated and the test F-Measures were averaged. As shown in Figure S17, when σ_R increases to 5%, the F-Measure is still 1. Because the experimentally observed σ_R is within 5% (Figure S15), its influence on the performance of the FE-PS-NET-based edge detection is thus negligible.

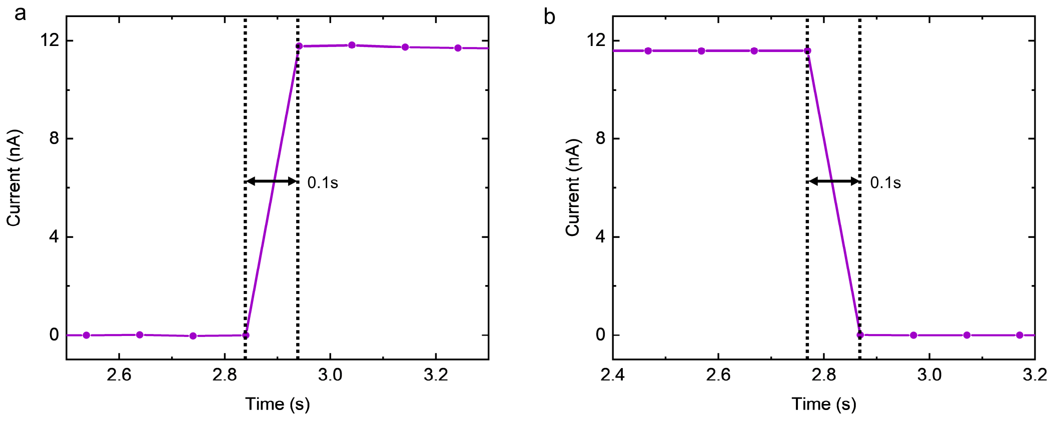

Figure S18. Transient current responses upon (a) illumination ON and (b) illumination OFF.

The photocurrent generation and decay times are measured to be ~100 ms, which reach the time resolution limit of our measurement system. Previous studies[5,6] using pulsed laser and high-speed oscilloscope demonstrated that the photocurrent generation and decay times could be below 1 ns.

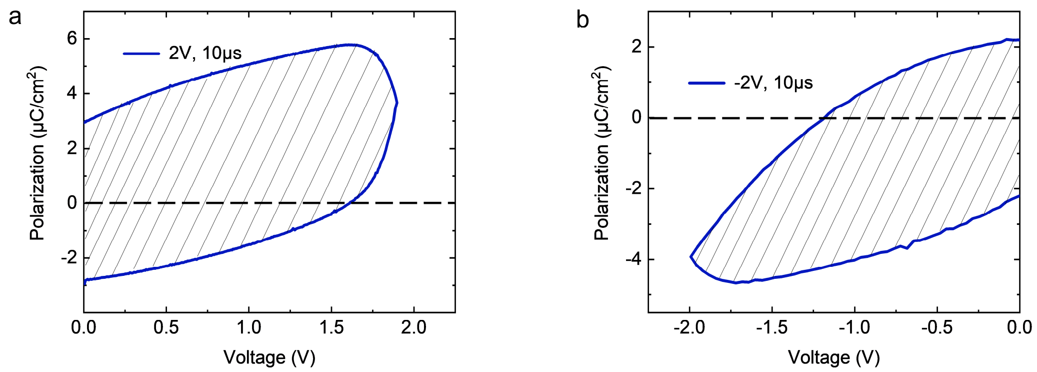

Figure S19. Typical monopolar *P-V* hysteresis loops measured with (a) 2 V/10 μs and (b) -2 V/10 μs pulses.

The energy consumption is calculated as follows:

$$\Delta E = \int_{t_1}^{t_2} VIdt = \int_{P_1}^{P_2} VdP \cdot A_{\mathrm{ele}} , \qquad \text{(S1)}$$

where V is the applied voltage, I is the total current including contributions from polarization switching, leakage current, and linear dielectric response, t is the time, P is the polarization, and A_{ele} is the electrode area. The total I is measured and integrated into P in the conventional *P-V* measurement. The total energy consumption therefore well corresponds to the product of the area within the *P-V* loop and A_{ele}. Using the shaded areas shown in Figure S19, the energies consumed by the positive and negative writing processes are calculated to be ~3.6 nJ and ~2.5 nJ, respectively, giving rise to an average energy consumption of ~3.1 nJ.

Supplementary Note 1. Reasons for choosing ~120 nm PZT film for this study.

In our previous work[7], we conducted a systematic study on the thickness-dependent photovoltaic properties of PZT films. We found that the PZT film with thickness of ~120 nm exhibited higher photovoltaic performance than thicker films. However, when further reducing the film thickness, it was difficult to obtain good ferroelectricity and photovoltaic switchability in the PZT films with large top electrodes (~200 μm in diameter) because of the leakage issue. We have therefore chosen the PZT film with the specific thickness of ~120 nm for this study. However, because the growth conditions have been modified, the ferroelectric and photovoltaic properties of the ~120 nm PZT films in our previous work and this work are not exactly the same.

Supplementary Note 2. Method for kernel weight mapping.

When mapping the kernel weights to the photoresponsivities, the target photoresponsivity mapped from √2 (-√2) was ~0.31 (~-0.31) mA/W, out of the accessible range of ~-0.22 to ~0.22 mA/W. To address this issue, the actual photoresponsivity of the FE-PS was set to be ~0.22 mA/W (or ~-0.22 mA/W) while the optical power applied to this FE-PS was scaled by a factor of √2; hence, the output current remained almost unchanged and the "nominal" photoresponsivity in such case may be regarded as ~0.31 mA/W (or ~-0.31 mA/W).

Supplementary Note 3. Latency estimation.

To highlight the low latency of the FE-PS-NET for image processing, a performance comparison was made between the FE-PS-NET and a conventional Von Neumann system comprising a camera, a memory, and a processor. A 10-million-pixel image showing one of the ten digits (‘0’ to ‘9’) was assumed as the image to be detected and processed. Here, the image referred to an illumination pattern, which was thus readily to be detected and processed. The image processing task was assumed to be the classification of this image into one of the classes ranging from ‘0’ to ‘9’.

A 10×10^7 ($M = 10$ and $N = 10^7$) FE-PS-NET was used for the simultaneous detection and processing of the image. The total latency mainly included two parts: the network latency and the peripheral circuit latency. For the network latency, as mentioned in the main text, the photocurrent generation time could be neglected compared with the RC time constant of the circuit, and the latter was considered to be mainly responsible for the network latency. To estimate it, each FE-PS was assumed to have a small area of ~1 μm^2 and a resistance of ~5.7×10^{10} Ω.[8] In addition, the thickness and dielectric constant of the PZT film in the FE-PS were assumed to be ~120 nm and ~180,[9] respectively, giving rise to a capacitance of ~1.3×10^{-14} C for the FE-PS. Using these parameters of FE-PS, we simulated a 10×10^7 FE-PS-NET (divided into multiple tiles) with current-sense-amplifiers (for current detection) in the NeuroSim simulator.[10] The network latency was calculated to be ~2.6 μs. For the peripheral circuit latency, we mainly considered the latency of the CMOS-based circuit of sigmoid activation function. It was calculated to be ~2.3 ns by using the NeuroSim simulator, much shorter than the network latency. The total latency was thus estimated to be ~2.6 μs for the FE-PS-NET.

In terms of the Von Neumann system, the image capture time consumed by a 10-megapixel camera (assuming a HP RJ23W3BA0KT CCD camera) was estimated to be ~20 ms. The image storage time was not estimated due to the lack of data. For the image processing, a NVIDIA GTX1650 GPU (memory size: 4 GB; memory clock: 8 GHz) was used. By running the matrix-vector multiplication involved in the classification task on the GPU, the image processing time was estimated to be ~0.77 ms. The total latency of the

Von Neumann system was thus at least ~20.77 ms, which was four orders of magnitude longer than that of the FE-PS-NET.

Certainly, the above latency estimations are quite preliminary, and further research is warranted to experimentally measure the latency of a practical large-scale FE-PS-NET.

References

1. Liu, H., Yang, P., Yao, K., & Wang, J. Twinning rotation and ferroelectric behavior of epitaxial $BiFeO_3$ (001) thin film. *Appl. Phys. Lett.* **96**, 012901 (2010).

2. Khan, A. I., Marti, X., Serrao, C., Ramesh, R., & Salahuddin, S. Voltage-controlled ferroelastic switching in Pb $(Zr_{0.2}Ti_{0.8})$ O_3 thin films. *Nano Lett.* **15**, 2229-2234 (2015).

3. Pintilie, L. et al. Polarization induced self-doping in epitaxial Pb $(Zr_{0.20}Ti_{0.80})$ O_3 thin films. *Sci. Rep.* **5**, 1-14 (2015).

4. Walker, D., Thomas, P. A., & Collins, S. P. A comprehensive investigation of the structural properties of ferroelectric $PbZr_{0.2}Ti_{0.8}O_3$ thin films grown by PLD. *Phys. Status Solidi (a)* **206**, 1799-1803 (2009).

5. Li, J. K., Ge, C., Jin, K. J., Du, J. Y., Yang, J. T., Lu, H. B., & Yang, G. Z. Self-driven visible-blind photodetector based on ferroelectric perovskite oxides. *Appl. Phys. Lett.* **110**, 142901 (2017).

6. Xing, J., Guo, E. J., Dong, J., Hao, H., Zheng, Z., & Zhao, C. High-sensitive switchable photodetector based on $BiFeO_3$ film with in-plane polarization. *Appl. Phys. Lett.* **106**, 033504 (2015).

7. Tan, Z. et al. Thinning ferroelectric films for high-efficiency photovoltaics based on the Schottky barrier effect. *NPG Asia Mater.* **11**, 20 (2019).

8. Fan, H. et al. Large electroresistance and tunable photovoltaic properties of ferroelectric nanoscale capacitors based on ultrathin super-tetragonal $BiFeO_3$ films. *J. Mater. Chem. C* **5**, 3323-3329 (2017).

9. Pintilie, L., Vrejoiu, I., Hesse, D., LeRhun, G., & Alexe, M. Ferroelectric polarization-leakage current relation in high quality epitaxial Pb (Zr, Ti) O_3 films. *Phys. Rev. B* **75**, 104103 (2007).

10. Chen, P. Y., Peng, X., & Yu, S. NeuroSim: A circuit-level macro model for benchmarking neuro-inspired architectures in online learning. *IEEE Transactions on Computer-Aided Design of Integrated Circuits and Systems* **37**, 3067-3080 (2018).
